# New Metallophthalocyanines Bearing 2-Methylimidazole Moieties—Potential Photosensitizers against *Staphylococcus aureus*

**DOI:** 10.3390/ijms23115910

**Published:** 2022-05-25

**Authors:** Marcin Wierzchowski, Daniel Ziental, Dawid Łażewski, Artur Korzanski, Agnieszka Gielara-Korzanska, Ewa Tykarska, Jolanta Dlugaszewska, Lukasz Sobotta

**Affiliations:** 1 Chair and Department of Chemical Technology of Drugs, Poznan University of Medical Sciences, Grunwaldzka 6, 60-780 Poznan, Poland; mwierzch@ump.edu.pl (M.W.); lazewskidawid@gmail.com (D.Ł.); agk@ump.edu.pl (A.G.-K.); etykarsk@ump.edu.pl (E.T.); 2 Chair and Department of Inorganic and Analytical Chemistry, Poznan University of Medical Sciences, Rokietnicka 3, 60-806 Poznan, Poland; dziental@ump.edu.pl; 3 Department of Chemistry, Adam Mickiewicz University, Uniwersytetu Poznanskiego 8, 61-614 Poznan, Poland; artur_ko@amu.edu.pl; 4 Chair and Department of Genetics and Pharmaceutical Microbiology, Poznan University of Medical Sciences, Rokietnicka 3, 60-806 Poznan, Poland; jdlugasz@ump.edu.pl

**Keywords:** phthalocyanines, singlet oxygen, PDT, PACT, metronidazole

## Abstract

Newly developed tetra- and octasubstituted methimazole-phthalocyanine conjugates as potential photosensitizers have been obtained. Synthesized intermediates and final products were characterized by the MALD-TOF technique and various NMR techniques, including 2D methods. Single-crystal X-ray diffraction was used to determine the crystal structures of dinitriles. The studied phthalocyanines revealed two typical absorption bands—the Soret band and the Q band. The most intense fluorescence was observed for octasubstituted magnesium(II) phthalocyanine in DMF (Φ_FL_ = 0.022). The best singlet oxygen generators were octasubstituted magnesium(II) and zinc(II) phthalocyanines (Φ_∆_ 0.56 and 0.81, respectively). The studied compounds presented quantum yields of photodegradation at the level between 10^−5^ and 10^−6^. Due to their low solubility in a water environment, the liposomal formulations were prepared. Within the studied group, octasubstituted zinc(II) phthalocyanine at the concentration of 100 µM activated with red light showed the highest antibacterial activity against *S. aureus* equal to a 5.68 log reduction of bacterial growth.

## 1. Introduction

**Figure 1:**
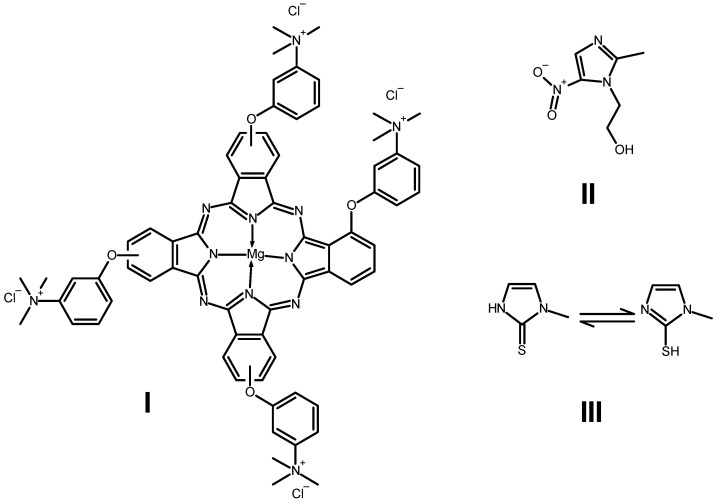
Structures of RLP068 (**I**), metronidazole (**II**) and methamizole (**III**).

Phthalocyanines are a dye family and are composed of four isoindole units linked with aza-methine bridges. These dyes reveal interesting properties potentially useful in nonlinear optics [1,2], solar energy conversion [3,4] and the medical field for photodynamic therapy (PDT) [5,6,7,8]. Photodynamic therapy is based on the interaction of molecular oxygen, a photosensitizer (PS) and light to produce highly active species—singlet oxygen. Singlet oxygen is responsible for cancer and bacteria cells combating [7,9,10]. Photodynamic therapy is becoming increasingly important as a beneficial remedy in treating bacterial infections [11,12,13,14]. More and more antibiotics are currently partially or entirely ineffective in the fight against common bacteria. Many scientists are indicating that we are on the threshold of the so-called “post-antibiotic era”. Due to the resistance of many bacteria to commonly used drugs, photodynamic therapy reveals many desirable features [15,16]. First of all, bacteria have not yet developed resistance to reactive oxygen species. Secondly, reactive oxygen species (ROS) do not attack any specific cell organelles [17]. The nonspecific target makes it difficult for pathogens to create defense mechanisms. Moreover, the lifetime of ROS is relatively short. Therefore bacteria are not able to develop resistance even after multiple uses of PDT [18]. Recently, there have been many promising studies on the efficacy of phthalocyanines in photodynamic antimicrobial chemotherapy (PACT) [19,20,21,22,23,24]. In 2020, Monami et al. published promising results of a clinical trial in which patients with diabetic foot ulcers underwent photodynamic therapy using a phthalocyanine derivative (**I**, RLP068, Figure 1) as a PS [19]. Additionally, Mannucci et al. studied the same phthalocyanine activity against bacteria-colonizing diabetic foot ulcers. In both experiments, a significant decrease in the number of bacteria-colonizing wounds and faster healing of ulcers were observed [24]. It is worth emphasizing that the tested phthalocyanines did not show any harm to mammalian cells [21].

Imidazole belongs to azole heterocyclic compounds, characterized by two nitrogen atoms present at positions 1 and 3. Compounds containing the imidazole group have been interesting as new potential drugs in the treatment of many diseases. They have a broad spectrum of biological activity: anti-inflammatory, analgesic, antioxidant, antiulcer, antidiabetic and anticancer. However, one of the most interesting properties of imidazole derivatives is their antibacterial activity [25]. Currently, there are registered several antimicrobials containing an imidazole ring in their structure: Ketoconazole, Metronidazole, Ornidazole, Azomycin, Oxiconazole and Miconazole. The mechanism of action of these drugs is not fully understood, but it may be based, in part, on the release of K^+^ ions in bacteria cells [25]. There are numerous studies on the development of entirely new compounds with antibacterial properties based on the imidazole ring [26,27,28,29]. In recent years, it has been observed that the alkalinity of imidazole may contribute to a better solubility of compounds in water at a lower pH, which may facilitate their use in medicine and increase the bioavailability [30]. The biological activity of the imidazole-containing compounds can vary significantly. The type and length of the substituents, as well as their position in the ring structure, are crucial for the antibacterial potential [28].

Moreover, as proven in research, azoles are inhibitors of enoyl acyl carrier protein reductase (FabI). This enzyme is an exciting molecular target for antibiotics. This may also be the reason why some azoles are active against methicillin-resistant *Staphylococcus aureus* strains [31]. In the presented work, an attempt was made to combine two therapeutic approaches. The unique ability of phthalocyanines to generate ROS and, thus, damage the bacterial cell structures was used. Moreover, the structure of the PS was modified by introducing an imidazole ring substituted with a methyl group. As reported so far, a substitution at the 2-position of the imidazole ring may increase the antimicrobial compound activity [32]. The presented strategy may prove to be an attractive solution in the future of designing PSs in PACT. Additionally, the presence of imidazole in the structure facilitates the interaction of the compound with biological molecules, including receptors, DNA and enzymes [32].

A particularly interesting imidazole-like compound is the metronidazole (**II**, Figure 1). This molecule provides a broad spectrum of useful properties for the treatment of infections [33,34], inflammations [35] and cancer [36].

Therefore, we decided to introduce a imidazole ring to the macrocycle and check of the antibacterial potential of new PSs. For the reaction, methamizole (**III**, Figure 1) as a substituent was used. The choice of **III** as a substrate allowed connection formation between phthalocyanine (Pc) and the imidazole ring via sulphide bridges.

## 2. Results

### 2.1. Synthesis

**Scheme 1:**
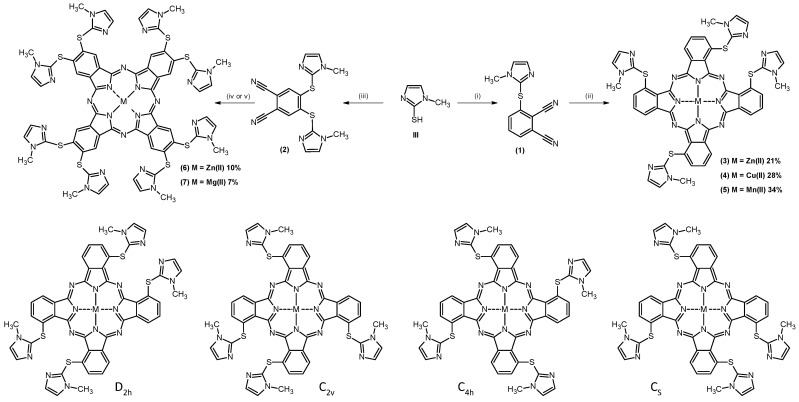
Synthesis of new compounds **1**–**7**. Reagents (**1**, **2**, **III**) and conditions and yields: (**i**) 3-nitrophthalonitrile, anhydrous K_2_CO_3_, DMF, 80 °C, 24 h, 79%; (**ii**) appropriate salt (Zn(OAc)_2_ for Zn, CuCl_2_ for Cu and MnCl_2_ ∙ 4 H_2_O for Mn), DBU, *n*-pentanol, 140 °C, 24 h; (**iii**) 4,5-dichlorophthalonitrile, anhydrous K_2_CO_3_, DMF, 80 °C, 24 h, 81%. (**iv**) Zn(OAc)_2_, DBU, *n*-pentanol, 140 °C, 24 h; (**v**) Mg, *n*-butanol, cat. I_2_, reflux 3 h, then, **2**, reflux, 24 h. The possible structural isomers of compounds **3**–**5** with symmetrical D_2h_, C_4h_, C_2v_ and C_s_. Structures of octasubstituted phthalocyanines **6** and **7**.

The synthesis of the new methimazole-phthalocyanine conjugates **3–7** was obtained in two different synthetic pathways, as shown in Scheme 1. The synthesis of tetrasubstituted compounds (**3**–**5**) was preceded by obtaining the conjugate of methimazole and 1,2-dicyanobenzene 1 by a nitro group displacement reaction with 3-nitrophtalonitrile. This process was performed in the presence of a base (anhydrous potassium carbonate), solvent *N,N*-dimethylformamide (DMF), under temperature 80 °C and lasted 24 h. Product 1—2-[(2,3-dicyanophenyl)thio]-1-methyl-1*H*-imidazole was isolated from the reaction mixture and purified by crystallization with a yield of 79%. In the second synthetic path, 4,5-bis[(1-methyl-1*H*-imidazol-2-yl)thio]-1,2-dicyanobenzene (**2**) was obtained by the substitution of chlorine atoms in 4,5-dichlorophthalonitrile with sulfur atoms of the “thiol” tautomeric form of methimazole. As with the synthesis of compound **1**, the process requires the presence of anhydrous K_2_CO_3_, DMF as a solvent, a temperature of 80 °C and time of 24 h. Compound **2** was obtained with a yield of 81%. All macrocyclization reactions were performed under ambient gas (N_2_) and were protected from exposition to light to avoid the decomposition of photoactive reaction products. These cyclotetramerization reactions of dinitriles **1** and **2** were performed in two different conditions. Compounds **3**–**6** were synthesized by adapting the procedure described by Vacus et al. [37]. The cyclotetramerization reaction of 3-substituted phthalonitriles resulted in the presence of four structural isomers shown in Scheme 1: D_2h_, C_4h_, C_2v_ and C_s_. The observed ratio of these isomers obtained was 1:1:2:4, respectively [38]. In our study, we observed small amounts of D_2h_, C_4h_ and C_2v_, but only the main product with symmetry C_s_ was isolated for compounds **3**–**5**. The macrocyclization reaction of phthalonitrile **2** was carried out in *n*-pentanol in the presence of appropriate salt (zinc(II) acetate for **3** and **6**, copper(II) chloride for **4** and manganese(II) chloride tetrahydrate for **5**) and 1,8-diazabicyclo[5.4.0]undec-7-ene (DBU) as a base for 24 h at 130 °C. The yield of reactions for tetrasubstituted compounds was in the range of 21–34%, whereas a significantly lower yield was observed for octasubstituted compounds **6** and **7**—only 10% and 7%, respectively. The dinitrile **2** was found to be reactive under the normal macrocyclization conditions instead (a suspension of magnesium n-butoxide in refluxing n-butanol, possessed by reaction magnesium turnings with alcohol in the presence of catalytic amounts of iodine) [39]. Macrocyclic compounds **3**, **4**, **6** and **7** were purified on silica gel by flash column chromatography in the normal and reversed-phase systems and on cross-linked dextran gel (Sephadex) by size-exclusion chromatography. Compound **5** was purified by multiple crystallizations from chloroform and ethanol. HPLC analyses confirmed the purity of macrocyclic compounds **3**–**7** at a level above 95%.

### 2.2. X-ray Diffraction Studies

**Figure 2:**
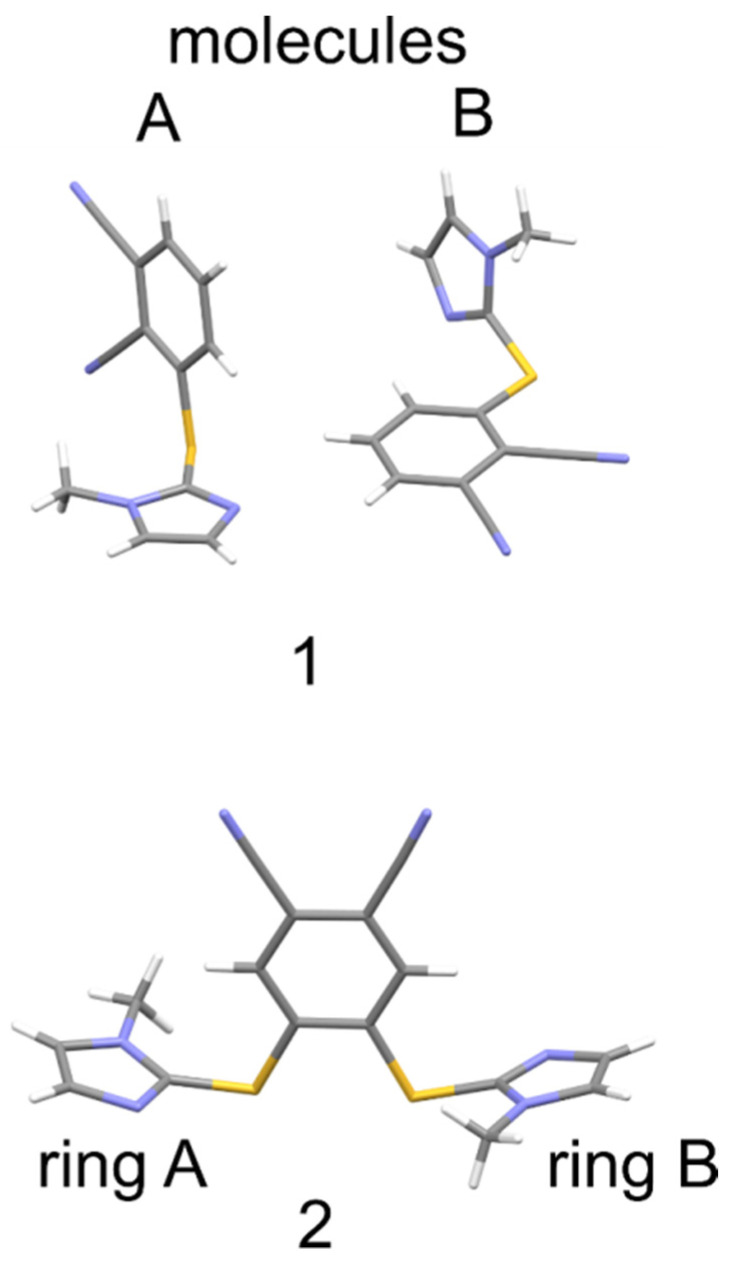
Crystal structures of **1** and **2**.

The crystal structures of **1** and **2** were determined by single-crystal X-ray diffraction. Both crystals were grown from ethanol by slow evaporation of the solution. Crystal structures **1** and **2** are triclinic with the P$\bar{1}$ space group. The asymmetric unit of **1** contains two molecules, while only one molecule is present in the asymmetric unit of **2**. Atom labeling of **1** and **2** is shown in Figure S1 (Supplementary Data) and the molecular structure in Figure 2. X-ray data analysis revealed that the imidazole rings were almost perpendicular to the phenyl rings. In **1**, the dihedral angles between five- and six-membered rings were 84.98(5)° for molecule 1A and 78.43(5)° for molecule 1B. The corresponding angles in structure **2** were 88.99(6)° and 83.73(5)° for the imidazole rings A and B, respectively. However, the imidazole methyl groups faced opposite sides of the phenyl ring (Figure 2). In both crystal structures, weak interactions of C-H···N and C-H···S types connected the molecules in a three-dimensional network (Table 1).

### 2.3. MALDI-TOF Mass Spectrometry

**Scheme 2:**
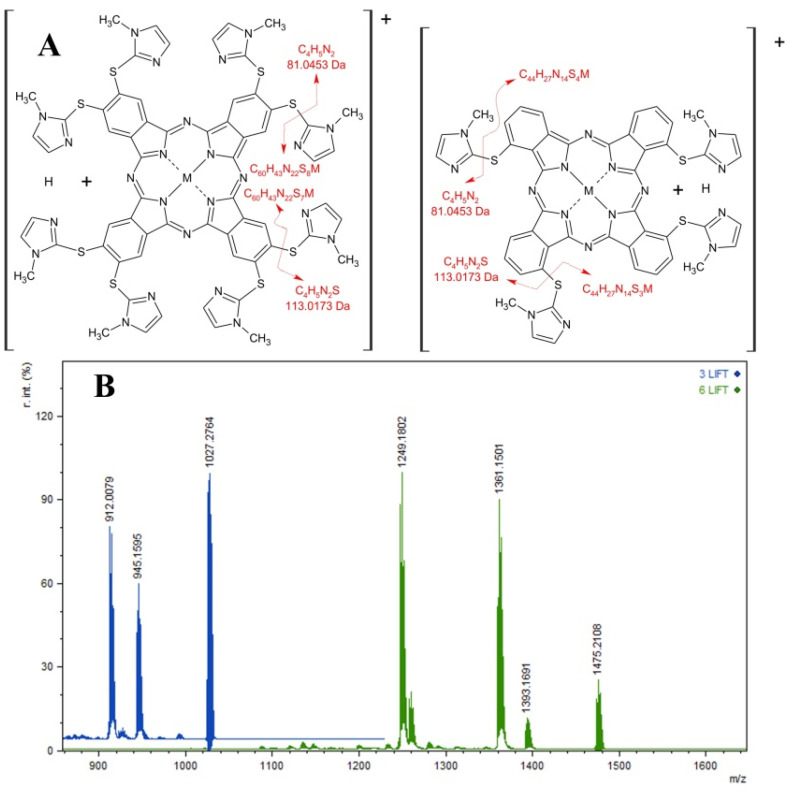
(**A**) Schematic representation of [M+H]^+^ fragmentation observed in MALDI-LIFT-TOF, arrows indicate the fragments formula. (**B**) LIFT spectra for compounds **3** and **6**.

The identity of new macrocyclic compounds was confirmed by the inter alia Matrix-Assisted Laser Desorption Ionization (MALDI) technique coupled with a Time-of-Flight analyzer (TOF). The path of decomposition generated in the ionization process quasimolecular ions [M+H]^+^ of new octa- and tetrasubstituted Pcs was studied. For this task the MALDI-LIFT-TOF technique was chosen, which was used successfully in the identification of macromolecular compounds such as proteins, peptides DNA and polymers [40,41,42]. Scheme 2A presents the observed fragmentation of parent ions [M+H]^+^. All macrocyclic compounds revealed the same pathway of fragmentation. In LIFT experiments, we observed peaks of daughter ions with decreased mass by 81 *m*/*z* and 113 *m*/*z*. This phenomenon is caused by the elimination of one 1-methylimidazole fragment or 1-methyl-2-thioimidazole fragments. In the case of octasubstituted Pcs, further fragmentation following the elimination of two 1-methyl-2-thioimidazole fragments was observed. Scheme 2B shows an example of the LIFT spectra for compounds **3** and **6**. Experimental data for compounds **4**, **5** and **7** in the Supplementary Data are shown.

### 2.4. NMR Study

**Figure 3:**
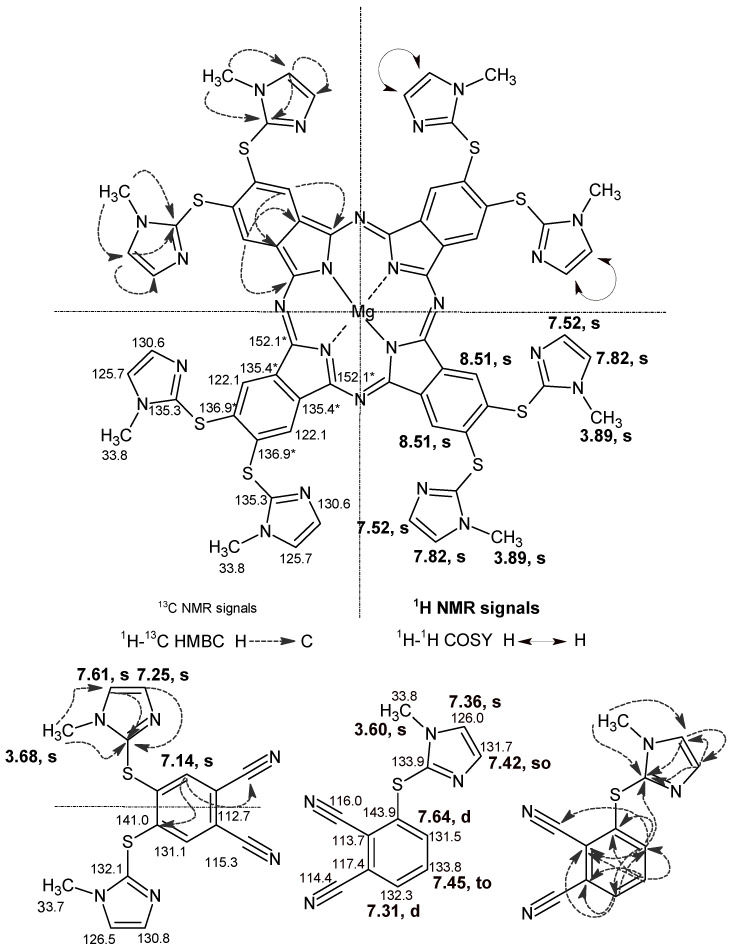
Signal annotations of **1**, **2** and **7** in DMSO-*d*_6_ from ^1^H, ^13^C, ^1^H–^1^H COSY, ^1^H–^13^C HSQC, ^1^H–^13^C HMBC spectra and the GIAO computational method (marked by an asterisk).

Spectra of phthalonitriles and Pcs were analyzed in a solution using various NMR techniques. Two-dimensional NMR techniques such as ^1^H-^1^H COSY (Correlation Spectroscopy), ^1^H-^13^C HSQC (Heteronuclear Single Quantum Correlation) and ^1^H-^13^C HMBC (Heteronuclear Multiple Bond Correlation) were used for the structure elucidation of the new compounds and annotation of the signals observed in ^1^H and ^13^C NMR. The recognized signals of exemplary compounds **1**, **2** and **7** are presented in Figure 3. The NMR experiments were performed in DMSO-*d*_6_ with the addition of pyridine or DBU in the case of macrocyclic compounds to avoid aggregation or precipitation. Signals of characteristic structural elements of phthalonitriles such as benzene ring protons at 7.64 ppm, doublet, 7.45 ppm triplet, 7.31 ppm, a doublet for compound **1** and 7.14 ppm for compound **2** were recognized. The presence of methimazole fragments confirmed three signals. First, singlet signals of the methyl group of compounds **1** and **2** were observed at 3.60 ppm and 3.68 ppm, respectively. However, the imidazole ring C4 proton signals were observed at 7.42 ppm for compound **1** and at 7.25 ppm for compound **2**. Proton signals of the C5 position were observed at 7.36 ppm (**1**) and 7.61 ppm (**2**). ^13^C NMR signals showed in Figure 3 indicated aromatic imidazole and benzene rings and cyano and methyl groups. Proton signals of new Pcs are similar to the phthalonitrile signals described above. Aromatic signals such as doublet 7.63 ppm, triplet 7.44 ppm, singlet 7.43 ppm, singlet 7.36 ppm and doublet 7.30 ppm represent Pc ring and imidazole substituents of compound **3**. The signal of a methyl group was observed at 3.59 ppm. The corresponding signals of aromatic protons of octasubstituted Pcs in a macrocyclic ring: C1, C4, C8, C11, C15, C18, C22 and C25 were observed as a singlet at 8.37 ppm for zinc(II) derivative **6**. Signals of the same protons of magnesium(II) compound **7** were observed at 8.51 ppm. Imidazole C5 protons for compounds **6** and **7** were observed as doublets at 7.79 ppm and 7.82 ppm, respectively. The presence of C4 imidazole protons confirmed doublets at 7.50 ppm (**6**) and 7.52 ppm (**7**). Figure 3 shows the complete annotation of ^13^C NMR signals of compound **7**. For signals observed at 136.9 ppm, 135.4 ppm and 152.1 ppm, 2D NMR techniques did not allow for an unambiguous signal assignment. In this case, a computational simulation was performed with the GIAO (Gauge-Independent Atomic Orbital) method with the DFT method (B3LYP functional and basis functions 6–31 Gdp).

### 2.5. Absorption and Emission

**Figure 4:**
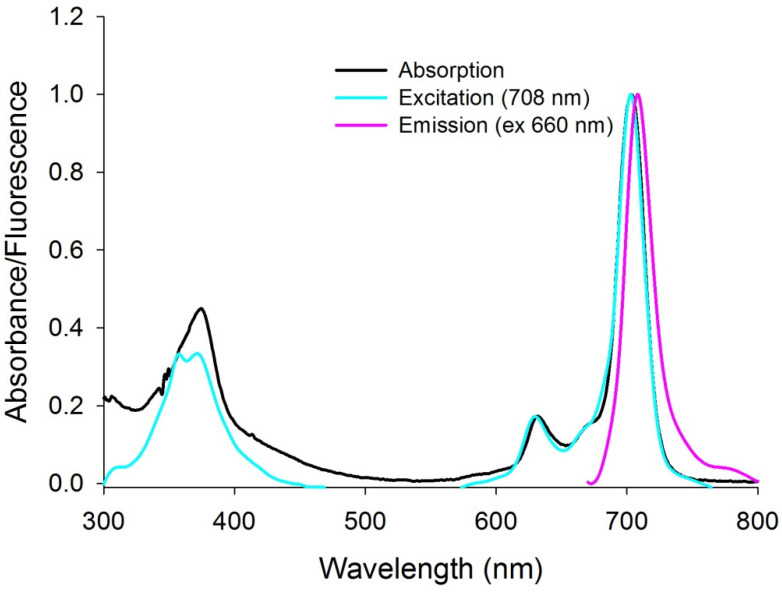
Absorption, excitation and emission spectra of **7** in DMF.

The studied Pcs revealed two typical absorption bands—at the range of 300–450 nm for the Soret band and in the range of 600–800 nm for the Q band (Figure 4). Among the studied group, the most intense fluorescence was observed for Pc **7**—0.022 in DMF. Recently, Zhang et al. published studies on magnesium(II) Pcs directly substituted with imidazole at peripheral and non-peripheral positions. These compounds revealed fluorescence quantum yields 0.21 and 0.31 for non-peripheral- and peripheral-substituted derivatives, respectively [43]. Compound **7**, compared to peripherally substituted Pc presented by Zhang et al., showed a dramatic drop in the fluorescence quantum yield about 14 times. This finding enabled us to conclude that the sulphide bridge in **7** quenched the fluorescence ability. Low emission was also reported by Güzel et al. for similar Pcs. They studied Pc derivatives substituted with thiadiazole moieties linked with the macrocyclic ring via the sulphide bridge [44].

### 2.6. Singlet Oxygen Formation

**Figure 5:**
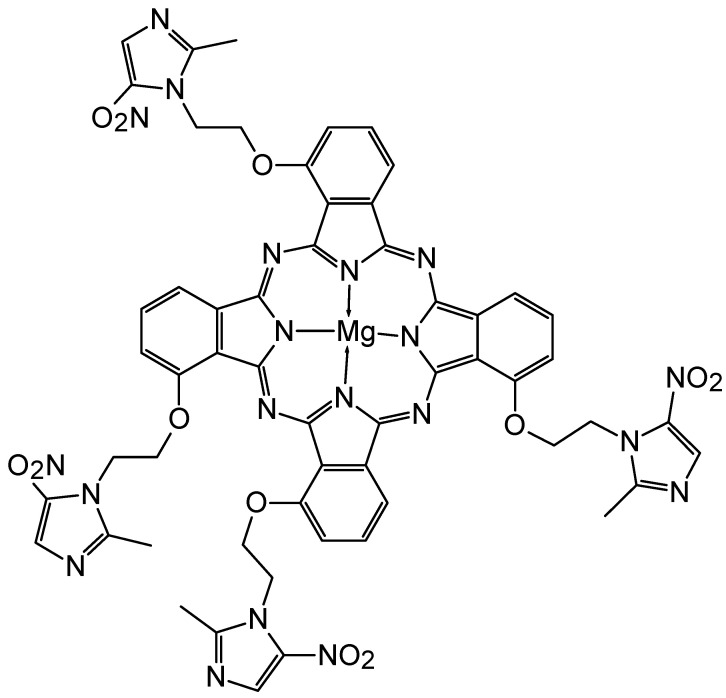
Structure of **IV**.

The key factor needed to provide a treatment effect is the reactive oxygen species. Singlet oxygen plays an essential role in this group [45]. Many authors have reported that singlet oxygen is the main tool for bacteria combat in the photodynamic process [15]. The best singlet oxygen generators were compounds **6** and **7** (Table 2). They were peripherally substituted Pcs with incorporated magnesium(II) and zinc(II) metal ions. In the past, we presented compounds similar to Pc **7**—Figure 5. The main difference between these photosensitizers is the position of the substitution; compound **7** is substituted peripherally and compound **IV** non-peripherally [46] with imidazole moieties. The change in the substitution position resulted in a dramatic increase of a singlet oxygen quantum yield value in DMSO forms 0.15 (**IV**) to 0.81 (**7**), whereas, in DMF, we observed comparable values. A similar tendency was reported by Baygu et al., who studied peripheral and non-peripheral thiosubstituted phthalocyanines [47].

Crucial for the singlet oxygen quantum yield seems to be an atom of the substituent attached to the macrocyclic ring. At the peripheral positions, thiosubstituted Pcs reveal high quantum yields measured in DMSO, whereas oxo-substituted ones have presented lower values [47,48,49,50,51,52]. The introduction of methimazole derivatives as substituents to the Pc ring via sulphide bridges results in a slight lowering of the Φ_Δ_ value in the case of compound **6** in comparison to the unsubstituted zinc(II) phthalocyanine (Table 2). The decrease in the singlet oxygen formation potential was due to the electron-donating substituents’ nature [53,54]. Interesting phenomena can be observed for **7** (MgPc) in comparison to **6** (ZnPc). Magnesium(II) phthalocyanine forms singlet oxygen more efficiently than zinc(II). This situation seems to be strange when the well-known “heavy atom effect” was analyzed. The Zinc(II) ion initiates spin-orbit coupling, which should result in a higher singlet oxygen formation [55]. Surprisingly, higher activity was detected for magnesium(II) phthalocyanine (**7**). On the other hand, we noticed that compound **3**—tetra-substituted zinc(II) phthalocyanine—in comparison with phthalocyanine **6**—the octa-substituted one—revealed a dramatically low singlet oxygen formation ability, with Φ_Δ_ = 0.05 in DMF and 0.09 in DMSO. Pcs bearing copper and chloromanganium ions are referred to as “open shells” due to the configurations of their orbital electrons. These kinds of macrocycles produce short-living excited states, and in consequence, low Φ_Δ_ values are observed—Table 2 [54,55].

### 2.7. Photostability

Another important issue in the new photosensitizer development is its stability upon irradiation. It was reported that Pcs after light irradiation decompose mainly according to the photobleaching pathway. This process leads to isoindole derivatives formation [59,60]. Isoindoles are compounds revealing their own activity. It was reported that the IC_50_ values of some isoindole derivatives against HeLa cells are placed between 140.60 and 383.82 µM [61]. Therefore, developed Pcs should present an optimal photostability. A photosensitizer is described as stable when Φ_P_ is around 10^−6^ and unstable when this parameter reaches values around 10^−3^ [62]. Studied here, compounds **3**, **5** and **6** presented quantum yields of photodegradation at the level of 10^−6^ (Table 2). Usually, the high stability of Pcs is linked with their low singlet oxygen generation quantum yields [57]. Pc **7**, the best singlet oxygen generator within the studied group, and its photodegradation quantum yield is one magnitude higher (10^−5^) than very stable derivatives **3**, **5** and **6** (10^−6^). Interestingly, the most unstable compound, **4**, with incorporated copper(II) ion into the center of the macro-ring, revealed low singlet oxygen formation ability. A high photodecomposition rate of copper(II) phthalocyanines has been reported before [54,63]. Some authors have also reported high photostability for zinc(II) phthalocyanine derivatives bearing down on their periphery heterocyclic azole moieties as substituents [51,62].

### 2.8. Photodynamic Activity against Bacteria

**Figure 6:**
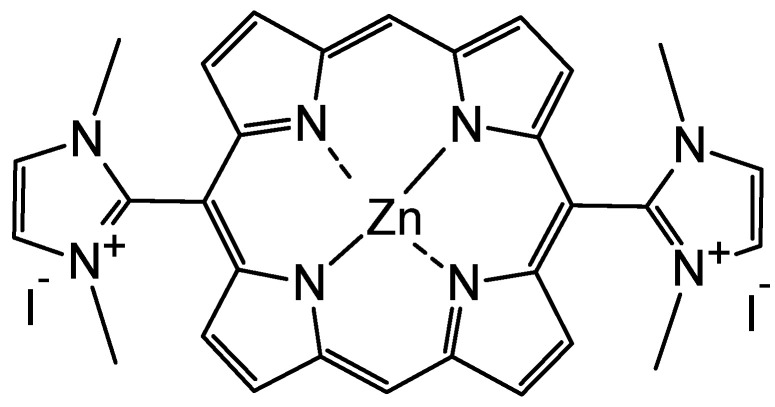
Structure of **V**.

*Staphylococcus aureus* is a Gram-positive pathogenic bacterium responsible for many health problems, including local skin infections and life-threatening systemic infections. The mentioned bacteria also cause chronic diseases such as osteomyelitis and otitis. Moreover, *S. aureus* produces proteins that interact with human plasma components, i.e., fibrinogen. The influence of this phenomenon on bacterial virulence has been extensively researched [64]. Therefore, in this paper, we examined the photodynamic *S. aureus* inactivation potential of synthetized Pcs. Due to the low solubility of the tested compounds in an aqueous environment, their liposomal formulations were prepared. The obtained liposomes revealed mean diameters in the range of 141–265 nm (Supplementary Data, Table S2). Within the studied group, derivative **6** at a concentration of 100 µM activated with red light with a maximal wavelength of 690 nm showed the highest antibacterial activity against *S. aureus* equal to a 5.68 log reduction of bacterial growth (Table 3). It should be highlighted that all studied Pcs have no activity without excitation with light (Supplementary Data, Table S3). This is a feature highly desirable for PACT. The activity of this compound can be assigned to a high singlet oxygen formation rate. Interestingly, magnesium(II) phthalocyanine **7**, despite its highest Φ_∆_ up to 0.81, revealed only a 0.29 log bacteria growth reduction at the same concentration as **6**. This phenomenon might result from a much easier protonation ability of magnesium(II) phthalocyanines, which was reported before. Protonated molecules upon irradiation reveal short-lived excited states, which lead to low quantum yields of fluorescence and singlet oxygen formation [65]. Recently, Vinagreiro et al. developed porphyrin V-bearing imidazole groups with high activity against bacteria with a reduction of the bacterial growth rate over 5 logs [66]. Thus, comparing the activities of **6** and **V** (Figure 6), it might be concluded that the imidazole group provided a high PACT activity of the PSs.

## 3. Materials and Methods

### 3.1. General

All reactions were performed using Radleys Heat-On™ heating system. Glassware was oven-dried under an argon atmosphere. All chemicals used as solvents and starting materials were purchased from commercial suppliers. All reaction mixture ingredients were used without further purification. However, dichloromethane was distilled before use. All solvents were isolated by rotary evaporation under reduced pressure at a temperature below 50 °C. A flash column chromatography was proceeded with Merck silica gel 60, particle size 40–63 µm, whereas thin-layer chromatography (TLC) was performed on silica gel Merck Kieselgel 60 F_254_ plates visualized with UV (λ_max_ 254 or 365 nm). Absorption spectra were measured on a Hitachi UV/Vis U-1900 and Shimadzu U-1900 spectrophotometers. IR spectra were recorded on a Jasco FT/IR-4600 spectrometer. Nuclear magnetic resonance spectra (NMR) were recorded on an Agilent DD2 800 spectrometer at 298 K. Chemical shifts (δ) were noted in parts per million (ppm) and orientated to the residual pyridine-d_5_ peak: δ_H_ 8.74, 7.58, 7.22 ppm, δ_C_ 150.35, 135.91, 123.87 ppm. Coupling constants (J) are shown in Hertz (Hz). The abbreviations s, t and m mean singlet, triplet, and multiplet, respectively. ^1^H and ^13^C signals were assigned to certain atoms based on ^1^H-^1^H COSY, ^1^H-^13^C HSQC and ^1^H-^13^C HMBC experiments.

### 3.2. Synthesis

#### 3.2.1. 2-[(2,3-Dicyanophenyl)thio]-1-methyl-1*H*-imidazole (**1**)

Anhydrous K_2_CO_3_ (3.19 g, 23.1 mmol), was added to a well-stirred slurry of methamizole (1.32 g, 11.6 mmol) and 3-nitrophtalonitrile (2.00 g, 11.6 mmol) in DMF (10 mL) and heated at 70–80 °C for 24 h. After cooling to room temperature, the reaction contents were poured into a water and ice mixture (100 mL) and left for 2 h. The resulting precipitate was isolated by filtration, washed three times with distilled water (3 × 50 mL) and crystallized from ethanol to give light beige-colored crystals of (**1**) (2.13 g, 79%). M.p. = 145–147 °C. R_f_ (10:1 CH_2_Cl_2_:CH_3_OH) 0.74. UV–Vis (CH**_2_**Cl**_2_**) λ_max_ [nm] (logε): 326 (3.39). ^1^H NMR (400 MHz, pyridine-*d*_5_) δ [ppm] 7.64 (d, J = 7,5 Hz, 1H), 7.45 (t, J = 8.0 Hz, 1H), 7.43 (s, 1H), 7.37 (s, 1H), 7.31 (d, J = 8,0 Hz, 1H), 3,60 (s, 3H). ^13^C NMR (100 MHz, pyridine-*d*_5_) δ [ppm] 143.9, 133.9, 133.8, 132.3, 131.7, 131.5, 126.0, 117.4, 116.0, 114.4, 113.7, 33.8. MS (ESI): *m*/*z* [M+H]^+^ 241. Anal. Cal. for C_12_H_8_N_4_S C(59.98), H(3.36), N(23.32), S(13.34). Found: C(59.85), H(3.52), N(22.92).

#### 3.2.2. 4,5-Bis[(1-methyl-1*H*-imidazol-2-yl)thio]-1,2-dicyanobenzene (**2**)

Methimazole (1.25 g, 10.9 mmol) and anhydrous K_2_CO_3_ (3.05 g, 22.1 mmol) were added to a well-stirred solution of 4,5-dichlorophthalonitrile (1.00 g, 5.0 mmol) in DMF (25 mL) for 24 h. After cooling to room temperature, the reaction contents were poured into a water and ice mixture (100 mL) and left for 2 h. The resulting precipitate was isolated by filtration, washed three times with distilled water (3 × 50 mL) and crystallized from ethanol to give light yellow-colored crystals (**2**) (1.56 g, 81%). M.p. = 237–238 °C. R_f_ (12:1 CH_2_Cl_2_:CH_3_OH) 0.73. UV–Vis (CH_3_OH) λ_max_ [nm] (logε): 260 (4.83). ^1^H NMR (500 MHz, DMSO-*d*_6_) δ [ppm] 7.61 (s, 2H), 7.25 (s, 2H), 7.14 (s, 2H), 3.68 (s, 6H). ^13^C NMR (126 MHz, DMSO-*d*_6_) δ [ppm] 141.0, 132.1, 131.1, 130.8, 126.5, 115.3, 112.7, 33.7. MS (ESI): *m*/*z* [M+H]**^+^** 353. Anal. Cal. for C_16_H_12_N_6_S_2_: C(54.53), H(3.43), N(23.85), S(18.12). Found: C(54.08), H(3.62), N(23.85).

#### 3.2.3. 1,8,15,25-Tetrakis[(1-methyl-1*H*-imidazo-2-yl)thio]phthalocyanine Zinc(II) (**3**)

Zinc(II) acetate (230 mg, 1.2 mmol) was added to a solution of phthalonitrile **1** (600 mg, 2.5 mmol) and 1,8-diazabicyclo[5.4.0]undec-7-ene (DBU) (210 μL, 1.4 mmol) in ***n***-pentanol (10 mL). The reaction mixture was vigorously stirred and heated at 140 °C for 24 h. Next, the solvent was evaporated under reduced pressure with toluene (2 × 50 mL), and the dry residue was purified by column chromatography on silica gel (CH_2_Cl_2_:CH_3_OH 15:1 next CH_2_Cl_2_:CH_3_OH 4:1). Evaporation of the collected eluates produced the dark green solid of **3** (134 mg, 21%). M.p. > 300 °C. R_f_ (CH_2_Cl_2_:CH_3_OH 15:1) 0.22. UV–Vis (CH_2_Cl_2_): λ_max_ [nm] (logε): 715 (4.40), 693 (4.36), 331 (4.15). ^1^H NMR (400 MHz, pyridine-*d*_5_) δ 7.63 (d, J = 7.6 Hz, 4H), 7.44 (t, J = 8.0 Hz, 4H), 7.43 (s, 4H), 7.36 (s, 4H), 7.30 (d, J = 8.2 Hz, 4H), 3.59 (s, 12H). ^13^C NMR (126 MHz, DMSO-*d*_6_) δ [ppm] 154.2, 153.5, 153.1, 152.4, 139.5, 139.4, 138.8, 135.6, 132.5, 132.4, 132.2, 132.0, 129.9, 125.8, 125.2, 120.4, 120.0, 54.9. HPLC purity 100.0% (Supplementary data). IR ν [cm^−1^]: 3112, 3061, 2927, 2852, 1705, 1622, 1564, 1453, 1406, 1384, 1313, 1277, 1224, 1144, 1100, 1037, 945, 892, 796, 757, 693, 588, 545.

#### 3.2.4. 1,8,15,25-Tetrakis[(1-methyl-1*H*-imidazo-2-yl)thio]phthalocyanine Copper(II) (**4**)

Copper(II) chloride (161 mg, 1.2 mmol) was added to a solution of phthalonitrile **1** (600 mg, 2.5 mmol) and 1,8-diazabicyclo[5.4.0]undec-7-ene (DBU) (210 μL, 1.4 mmol) in ***n***-pentanol (10 mL). The reaction mixture was vigorously stirred and heated at 140 °C for 24 h. Next, the solvent was evaporated under reduced pressure with toluene (2 × 50 mL), and the dry residue was purified by column chromatography on silica gel (CH_2_Cl_2_:CH_3_OH 15:1; next CH_2_Cl_2_:CH_3_OH 4:1). Evaporation of collected eluates produced the dark green solid of 4 (179 mg, 28%). M.p. > 300 °C. R_f_ (CH_2_Cl_2_:CH_3_OH 5:1) 0.45. UV–Vis (CH_2_Cl_2_): λ_max_ [nm] (logε): 705 (4.39), 654 (4.34), 325 (4.34). IR ν [cm^−1^]: 3083, 3030, 3928, 2850, 1641, 1586, 1465, 1440, 1320, 1240, 1204, 1155, 1105, 982, 742, 690. HPLC purity 95.0–100.0% (Supplementary Data).

#### 3.2.5. 1,8,15,25-Tetrakis[(1-methyl-1*H*-imidazo-2-yl)thio]phthalocyanine Manganese(II) (**5**)

Manganese(II) chloride tetrahydrate (238 mg, 1.2 mmol) was added to a solution of phthalonitrile **1** (600 mg, 2.5 mmol) and 1,8-diazabicyclo[5.4.0]undec-7-ene (DBU) (210 μL, 1.4 mmol) in *n*-pentanol (10 mL). The reaction mixture was vigorously stirred and heated at 140 °C for 24 h. Next, the solvent was evaporated under reduced pressure with toluene (2 × 50 mL), and the dry residue was purified by recrystallization from CHCl_3_ and followed by recrystallization from ethanol. It gave a brown solid of **5** (216 mg, 34%). M.p. >300 °C. UV–Vis (CH_2_Cl_2_): λ_max_ [nm] (logε): 726 (5.53), 516 (4.91), 356 (5.29). IR ν [cm^−1^]: 3125, 2942, 1575, 1459, 1326, 1283, 1235, 1180, 1104. 1081, 1041, 914, 808, 766, 734, 672. HPLC purity 97.6–98.0% (Supplementary Data).

#### 3.2.6. 2,3,9,10,16,17,23,24-Octakis[(1-methyl-1*H*-imidazo-2-yl)thio]phthalocyanine Zinc(II) (**6**)

Zinc acetate (130 mg, 0.7 mmol) was added to a solution of phthalonitrile **2** (500 mg, 1.4 mmol) and 1,8-diazabicyclo[5.4.0]undec-7-ene (DBU) (200 μL, 1.4 mmol) in ***n***-pentanol (5 mL). The reaction mixture was vigorously stirred and heated at 140 °C for 24 h. Next, the solvent was evaporated under reduced pressure with toluene (2 × 50 mL), and the dry residue was purified by column chromatography on silica gel (CH_2_Cl_2_:CH_3_OH 4:1) and Sephadex G-25 (CH_3_OH). Evaporation of the collected eluates produced the dark green solid of **6** (52 mg, 10%). M.p. > 300 °C. R_f_ (CH_2_Cl_2_:CH_3_OH 4:1) 0.89. UV–Vis (CH_2_Cl_2_): λ_max_ [nm] (logε): 707 nm (5.66), 532 nm (4.47), 374 nm (5.54), 269 nm (5.74). ^1^H NMR (500 MHz, DMSO-*d*_6_) δ [ppm] 8.40 (s, 8H, phthalocyanine ring C1, C4, C8, C11, C15, C18, C22 and C25), 7.77 (s, 8H, imidazole, C5), 7.48 (s, 8H, imidazole C4), 3.86 (s, 24H, -CH3); ^13^C NMR (126 MHz, DMSO-*d*_6_) δ [ppm] 153.3, 138.9, 136.2, 133.2, 130.5, 125.7, 121.6, 33.9. MS (MALDI-TOF): *m*/*z* [M+H]**^+^** 1478.2. IR ν [cm^−1^]: 3207, 2943, 1723, 1593, 1507, 1485, 1452, 1399, 1369, 1333, 1276, 1157, 1111, 1081, 1059, 936, 771, 743, 694, 554. HPLC purity 97.6–100.0% (Supplementary Data).

#### 3.2.7. 2,3,9,10,16,17,23,24-Octakis[(1-methyl-1*H*-imidazo-2-yl)thio]phthalocyanine Magnesium(II) (**7**)

A rapidly stirred mixture of magnesium turnings (41 mg, 1.7 mmol), *n*-butanol (40 mL) and I_2_ (1 crystal) was heated under reflux for 3 h. After the mixture was cooled to room temperature, phthalonitrile **2** (600 mg, 1.7 mmol) was added, and the reaction mixture was heated under reflux for further 24 h. After being allowed to cool to room temperature, the reaction mixture was filtered through celite and evaporated to dryness under reduced pressure with toluene (3 × 15 mL). Product purification was performed by column chromatography on silica gel (CH_2_Cl_2_:CH_3_OH 8:1, CH_2_Cl_2_:CH_3_OH 4:1) and Sephadex G-25 (CHCl_3_). Evaporation of the collected eluates gave the dark green solid of **7** (42 mg, 7%). M.p. > 300 °C. R_f_ (CH_2_Cl_2_:CH_3_OH 8:1) 0.19. UV–Vis (CH_2_Cl_2_): λ_max_ [nm] (logε): 807 (4.78), 506 (4.28), 429 (4.43), 353 (4.51), 275 (4.64). ^1^H NMR (500 MHz, DMSO-*d*_6_) δ [ppm] 8.51 (s, 8H), 7.82 (s, 8H), 7.52 (s, 8H), 3.89 (s, 24H). ^13^C NMR (126 MHz, DMSO-*d*_6_) δ [ppm] 152.1, 136.9, 135.9, 135.4, 130.6, 125.9, 122.1, 33.8. MS (MALDI-TOF): *m*/*z* [M+H]**^+^** 1434.1. IR ν [cm^−1^]: 3125, 2925, 1721, 1593, 1508, 1450, 1398, 1367, 1335, 1275, 1156, 1105, 1079, 1056, 934, 882, 842, 775, 746, 746, 685, 559. HPLC purity 95.4–100.0% (Supplementary Data).

### 3.3. Single-Crystal X-ray Diffraction Studies

Crystals 1 and 2 were grown from ethanol by the slow evaporation technique. Reflection intensities were collected with the Oxford Diffraction SuperNova diffractometer using graphite-monochromated CuKα radiation at 293(2) K and 130(2) K for 1 and 2, respectively. Data were processed with the Agilent Technologies CrysAlis Pro software [67]. The structures were solved by direct methods (SHELXS [68] for 1 and Olex2 [69] for 2 and refined by the full-matrix least squares techniques based on F2 with SHELXL [68]. All non-H atoms were refined anisotropically. In 1, hydrogen atoms, except for the methyl groups, were located in the electron density maps, and their positions and isotropic displacement parameters were freely refined. The methyl hydrogen atoms in 1 and all hydrogen atoms in 2 were constrained to their calculated positions and were refined using a riding model with U_iso_(H) = 1.2U_eq_(C) or 1.5U_eq_ (methyl C). Interpretation of the results was performed using SHELXTL [68] and Mercury [70] programs. The crystal and refinement data are given in Table 4. The CIFs files have been deposited with the Cambridge Crystallographic Data Centre (www.ccdc.cam.ac.uk accessed on 5 March 2022) CCDC 2042390 and 2049132 for 1 and 2, respectively.

Computer programs: CrysAlis PRO, Agilent Technologies, Version 1.171.35.19 (release 27 October 2011 CrysAlis171. NET) (compiled 27 October 2011, 15:02:11), SIR2004 [71], SHELXL2014/7 [68] and Mercury [70].

### 3.4. Absorption and Emission

Absorption spectra of the studied compounds were recorded with a Shimadzu UV-160 spectrophotometer, and emission spectra were recorded using a JASCO 6200 spectrofluorometer at ambient temperature. The quantum yields of fluorescence were calculated with the method described earlier [56,72,73].

### 3.5. Singlet Oxygen Generation

The singlet oxygen formation quantum yield was determined in DMF and DMSO at ambient temperature. Experiments were performed with a comparative method described previously. As the chemical singlet oxygen scavenger was 1,3-diphenylisobenzofuran (Aldrich, Darmstadt, Germany), and as a reference was the unsubstituted zinc(II) phthalocyanine (Aldrich) [58,73,74,75].

### 3.6. Photostability Determination

The photodegradation quantum yields were determined in DMF and DMSO under aerobic conditions at an ambient temperature with the method described before [74,76,77,78].

### 3.7. Biological Activity

#### 3.7.1. Liposomes Preparation Procedure

POPC (1-palmitoyl-2-oleoyl-sn-glycero-3-phosphocholine) and DOTAP (***N***-[1-(2,3-dioleoyloxy)propyl]-***N,N,N***-trimethylammonium chloride, 25 mg/mL) (Avanti Polar Lipids Inc. Alabaster, AL, USA) dissolved in chloroform (Aldrich, Darmstadt, Germany) were mixed in a molar ratio of 8:2. In the next step, chloroform solutions of the studied compounds at the concentration of 1 mg/mL were added in an appropriate amount to achieve the final concentration in the vesicle of 200 µM. Then, the obtained mixture was evaporated under reduced pressure to give a thin lipid film. Next, the saline buffer was added and vortexed for 5 min. The size unification was performed by the extrusion of the mixture through the polycarbonate filter (100 or 200 nm). The size of the obtained vesicles was measured with NanoSight LM10 (Malvern Panalytical, Malvern, UK).

#### 3.7.2. Photodynamic Activity against Bacteria

The *Staphylococcus aureus* strain was purchased from the National Collection of Type Cultures (NCTC; *S. aureus* NCTC 4163) and cultured aerobically in BHI broth at 36 °C for 20 h. After this period, the bacteria were harvested by centrifugation (3000× *g* for 15 min) and resuspended in phosphate-buffered saline (PBS, pH = 7.0) to a final concentration of ca. 107 colony-forming units (CFU/mL). In a dark phase, aliquots of a microbial suspension were placed in the microtitration plate; then, solutions containing compounds incorporated in liposomal formulations were added and incubated for 20 min. The preincubation time was developed on the basis of previous experiments [53,76]. At the same time, adjusting the preincubation time was guided by the standard of not exceeding the estimated lag time for *S. aureus*, which is about 2 h [79]. This time should include the period of preincubation, radiation and other manipulations until plating. Photodynamic activity determination was performed in the light phase. This phase was made similarly to the dark one. The irradiation of the culture after an incubation time of 20 min with light at wavelength λ = 690 nm. It was provided a light dose of 30 J/cm^2^ applying high-power LED MultiChip Emitters (60 high-efficiency AlGaAs diode chips, Roithner LaserTechnik GmbH, Vienna, Austria). The light intensity and the concentration of PSs were selected using a modified checkerboard method [80]. In the study, a series of consecutive dilutions of PSs in decreasing concentrations was used, and they were subjected to light irradiation of increasing energy. The reverse scheme was utilized in the second series. In this way, theoretical breakpoints for the values of the PS concentrations and light intensity were established. Finally, bacterial suspension from each well was inoculated on tryptic soy agar (TSA) plates. After an incubation period (20 h at 36 °C), the viability of the bacteria was calculated by counting the number of CFUs.

## 4. Conclusions

Newly developed tetra- and octasubstituted methimazole-phthalocyanine conjugates as potential PSs were reported. Within the obtained compounds, the most promising photodynamic *Staphylococcus aureus* inactivation was 2,3,9,10,16,17,23,24-octakis[(1-methyl-1H-imidazo-2-yl)thio]phthalocyanine zinc(II) with the highest singlet oxygen quantum yield formation. Studied here, the compounds presented quantum yields of photodegradation at the level between 10^−5^ and 10^−6^, which enabled assigning them as photostable PSs. Unfortunately, the synthetized compounds revealed low solubility in the water environment; therefore, their liposomal formulation was prepared. The obtained liposomes revealed a mean diameter in the range of 141–265 nm, which enabled them to use this formulation in skin, intramuscular and subcutaneous administration. Zinc(II) phthalocyanine liposomal formulation at the concentration of 100 µM of PS activated with red light at 30 J/cm^2^ showed activity against *S. aureus* equal to a 5.68 log reduction of bacterial growth.

## Figures and Tables

**Table 1:** Geometry of selected C-H···N and C-H···S interactions in **1** and **2**.

*D*—H···*A*	*D*—H (Å)	H···*A* (Å)	*D*···*A* (Å)	*D*—H···*A* (°)
1				
C7A-H7A...S11B	0.97 (2)	2.99 (2)	3.938 (2)	168.0 (16)
C15A-H15A...N9A ^i^	0.97 (2)	2.66 (2)	3.306 (2)	124.6 (16)
C17B-H17E...N9B ^ii^	0.96	2.75	3.607 (2)	149.7
**2**				
C4A—H4A···N5B ^i^	0.95	2.69	3.612 (2)	162.5
C6A—H6A3···N7 ^ii^	0.98	2.57	3.331 (2)	134.9
C6B—H6B2···S1 ^iii^	0.98	2.85	3.608 (2)	134.4
C6B—H6B3···S2 ^iv^	0.98	2.78	3.688 (2)	154.2

Symmetry code(s) in **1**: (i) x, y, −1 + z; (ii) −x, 2 − y, −z. Symmetry code(s) in **2**: (i) x + 1, y − 1, z; (ii) −x + 2, −y + 1, −z + 1; (iii) −x + 1, −y + 1, −z; (iv) −x, −y + 1, −z.

**Table 2:** Fluorescence, photobleaching and singlet oxygen formation quantum yields for the studied phthalocyanines.

Compound	Solvent	Φ_FL_	10^6^Φ_P_	Φ_Δ_
**3**	DMF	0.002	2.95	0.05
	DMSO	0.001	4.47	0.09
**4**	DMF	-	83.40	0.04
	DMSO	0.001	39.80	0.08
**5**	DMF	-	3.21	0.03
	DMSO	-	2.46	0.03
**6**	DMF	0.003	4.84	0.13
	DMSO	0.001	2.97	0.56
**7**	DMF	0.022	14.20	0.11
	DMSO	0.005	13.30	0.81
**ZnPc**	DMF	0.200 [56]	10.2 [57]	0.56 [58]
	DMSO	0.170 [56]	3.5 [57]	0.67 [58]

**Table 3:** Photodynamic activity of the studied phthalocyanines against *Staphylococcus aureus* upon excitation with red light at the dose of 30 J/cm^2^.

Compound	3	4	5	6	7
Concentration (M)	log reduction in bacterial growth				
10^−4^	2.34	0.19	0.09	5.68	0.29
10^−5^	0.46	0.31	0.02	1.79	0.11

**Table 4:** Experimental details for 1 and 2.

	(1)	(2)
Crystal data		
Chemical formula	C_12_H_8_N_4_S	C_16_H_12_N_6_S_2_
*M*_r_	240.28	352.44
Crystal system, space group	$\mathrm{Triclinic},\;P\bar{1}$	$\mathrm{Triclinic},\;P\bar{1}$
Temperature (K)	293 (2)	130 (2)
*a*, *b*, *c* (Å)	8.7811 (4), 10.9839 (4), 12.3411 (3)	5.2154 (3), 10.751 (1), 14.794 (1)
α, β, γ (°)	80.390 (3), 84.655 (3), 89.429 (4)	103.532 (7), 98.293 (5), 93.655 (6)
*V* (Å^3^)	1168.48 (7)	793.94 (11)
*Z*	4	2
Radiation type	Cu *K*α α	Cu *K*α α
μ (mm^−1^)	2.31	3.13
Crystal size (mm)	0.5 × 0.2 × 0.15	0.40 × 0.10 × 0.05
Data collection		
Diffractometer	SuperNova, Single source at offset), Atlas	SuperNova, Single source at offset), Atlas
Absorption correction	Multi-scan	Multi-scan
*T*_min_, *T*_max_	0.276, 1.000	0.563, 1.000
No. of measured, independent and observed [*I* > 2σ(*I*)] reflections	13524, 4417, 4149	10291, 2792, 2641
*R*_int_	0.018	0.026
(sin θ/λ)_max_ (Å^−1^)	0.609	0.595
Refinement		
*R*[*F*^2^ > 2σ(*F*^2^)], *wR*(*F*^2^), *S*	0.035, 0.100, 1.06	0.034, 0.101, 1.05
No. of reflections	4417	2792
No. of parameters	347	219
H-atom treatment	H atoms treated by a mixture of independent and constrained refinement	H-atom parameters constrained
Δρ_max_, Δρ_min_ (e Å^−3^)	0.33, −0.30	0.31, −0.37

## Data Availability

Not applicable.

## Supplementary Materials

The following supporting information can be downloaded at https://www.mdpi.com/article/10.3390/ijms23115910/s1.
