# Supplementary material for: New Metallophthalocyanines Bearing 2-Methylimidazole Moieties—Potential Photosensitizers against Staphylococcus aureus
Source: Int J Mol Sci. 2022 May 25;23(11):5910. doi: 10.3390/ijms23115910 (PMC9180345; doi:10.3390/ijms23115910)
Supplement: Supplementary file 1 [file ijms-23-05910-s001.zip › ijms-1727677-supplementary.pdf]

# New metallophthalocyanines bearing 2-methylimidazole moieties – potential photosensitizers against bacteria

Marcin Wierzchowski[a], Daniel Ziental[b], Dawid Łażewski[a], Artur Korzanski[c], Agnieszka Gielara-Korzanska[a], Ewa Tykarska[a], Jolanta Dlugaszewska[d], Lukasz Sobotta[b*]

[a] *Chair and Department of Chemical Technology of Drugs, Poznan University of Medical Sciences, Grunwaldzka 6, 60-780 Poznan, Poland*
[b] *Chair and Department of Inorganic and Analytical Chemistry, Poznan University of Medical Sciences, Grunwaldzka 6, 60-780 Poznan, Poland*
[c] *Department of Chemistry, Adam Mickiewicz University, Uniwersytetu Poznanskiego 8, 61-614 Poznań, Poland*
[d] *Chair and Department of Genetics and Pharmaceutical Microbiology, Poznan University of Medical Sciences, Swiecickiego 4, 60-781 Poznan, Poland*

## Supporting Information


| Table of Contents | pages |
|---|---|
| **1. NMR data.........................................................................** | **2-11** |
| **2. Mass spectra data…...............................................................** | **12-17** |
| **3. IR data ..........................................................................** | **18-20** |
| **4. HPLC data.........................................................................** | **21-33** |
| **5. Computational data ...............................................................** | **34-35** |
| **6. Crystallographic data.............................................................** | **36** |
| **7. Liposomes……......................................................................** | **37** |
| **8. Dark activity of PSs against *Staphylococcus aureus*……………………………….** | **37** |

1. NMR data

$^{1}H$ NMR spectrum of **1** in pyridine-$d_5$

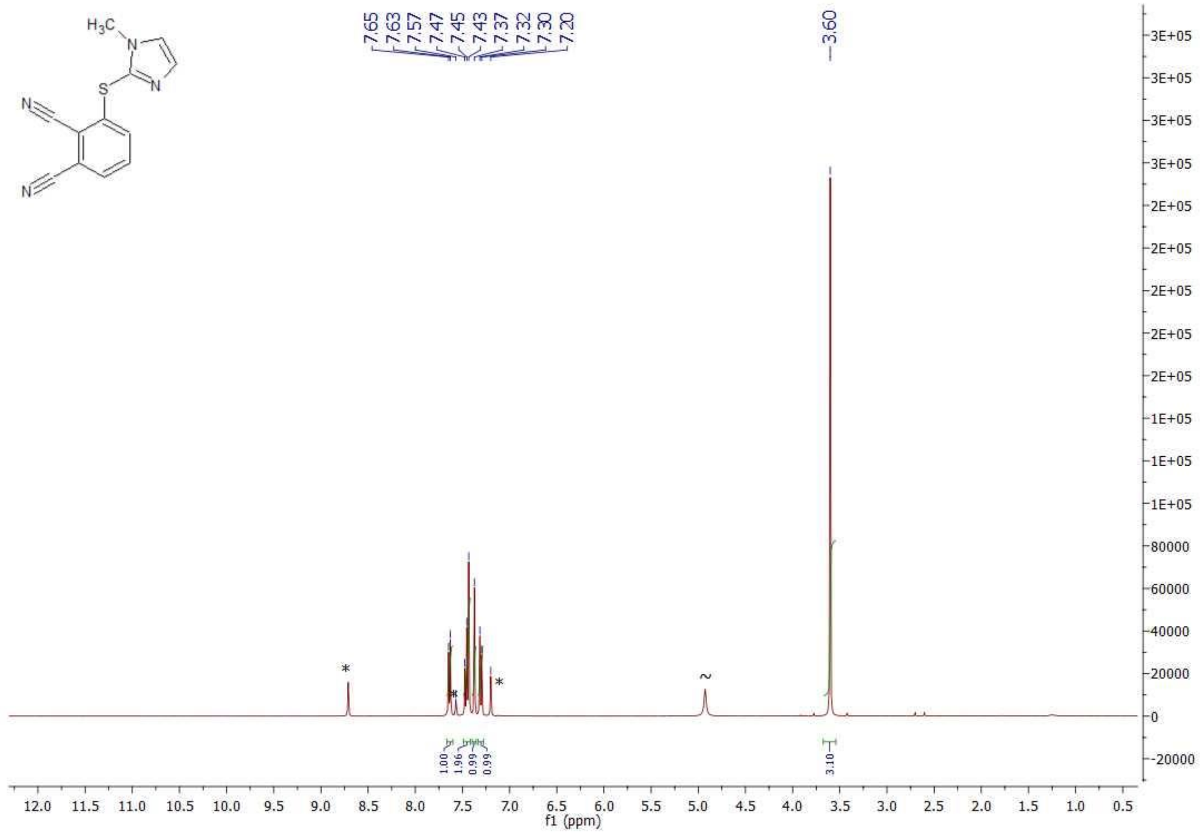


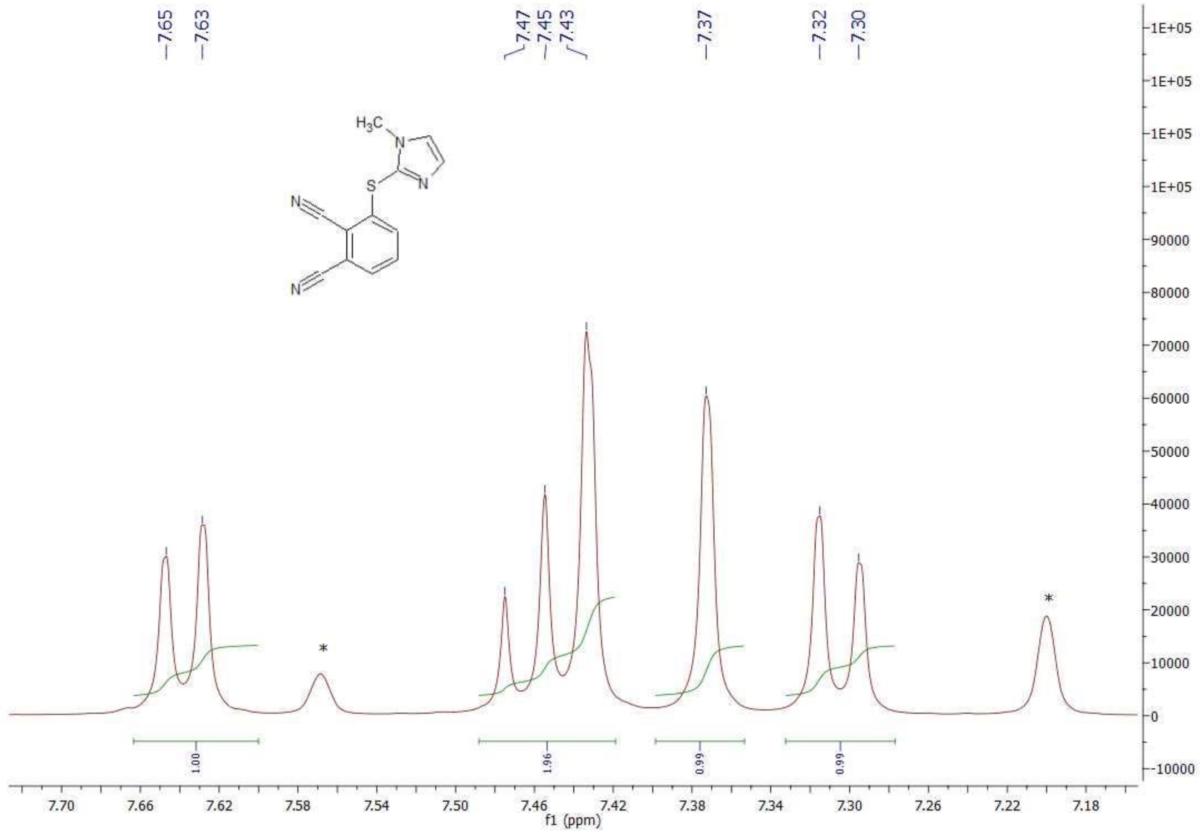

$^{13}C$ NMR spectrum of **1** in pyridine-$d_5$

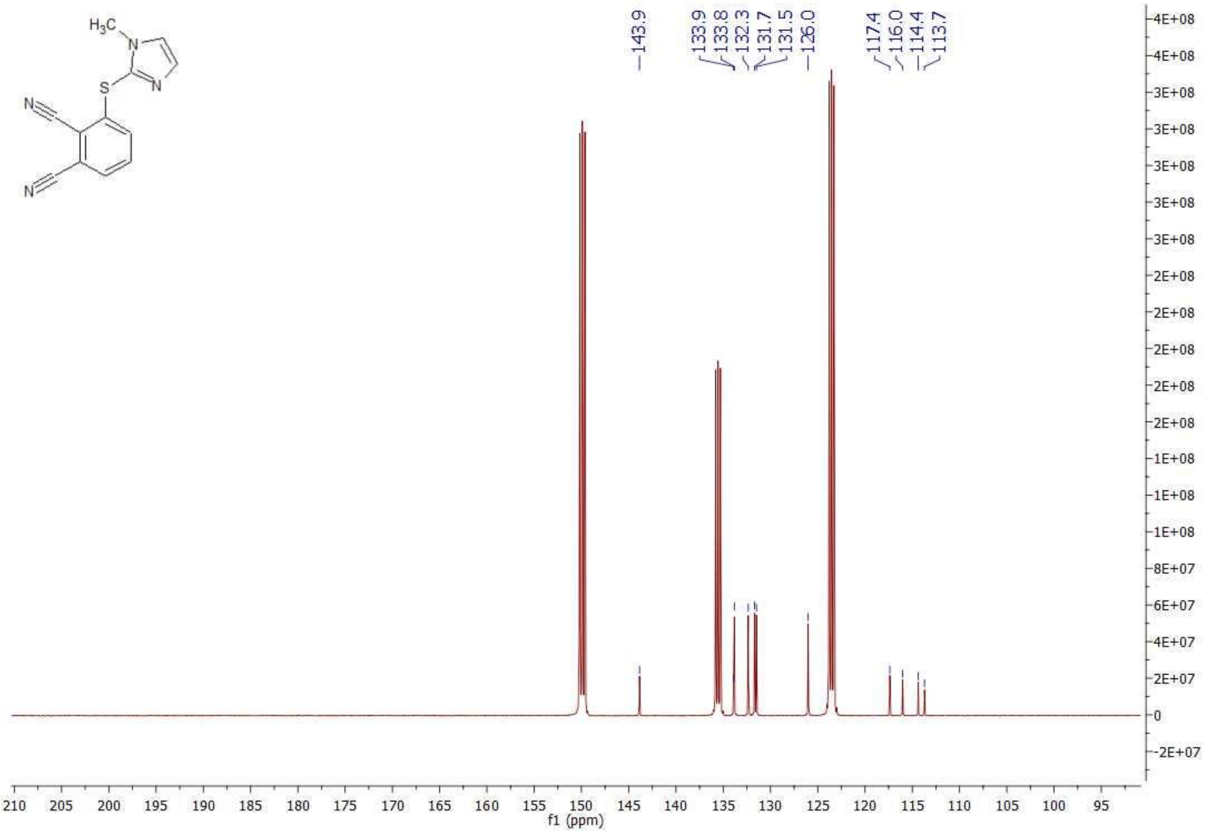


$^1H$-$^1H$ COSY spectrum of **1** in pyridine-$d_5$

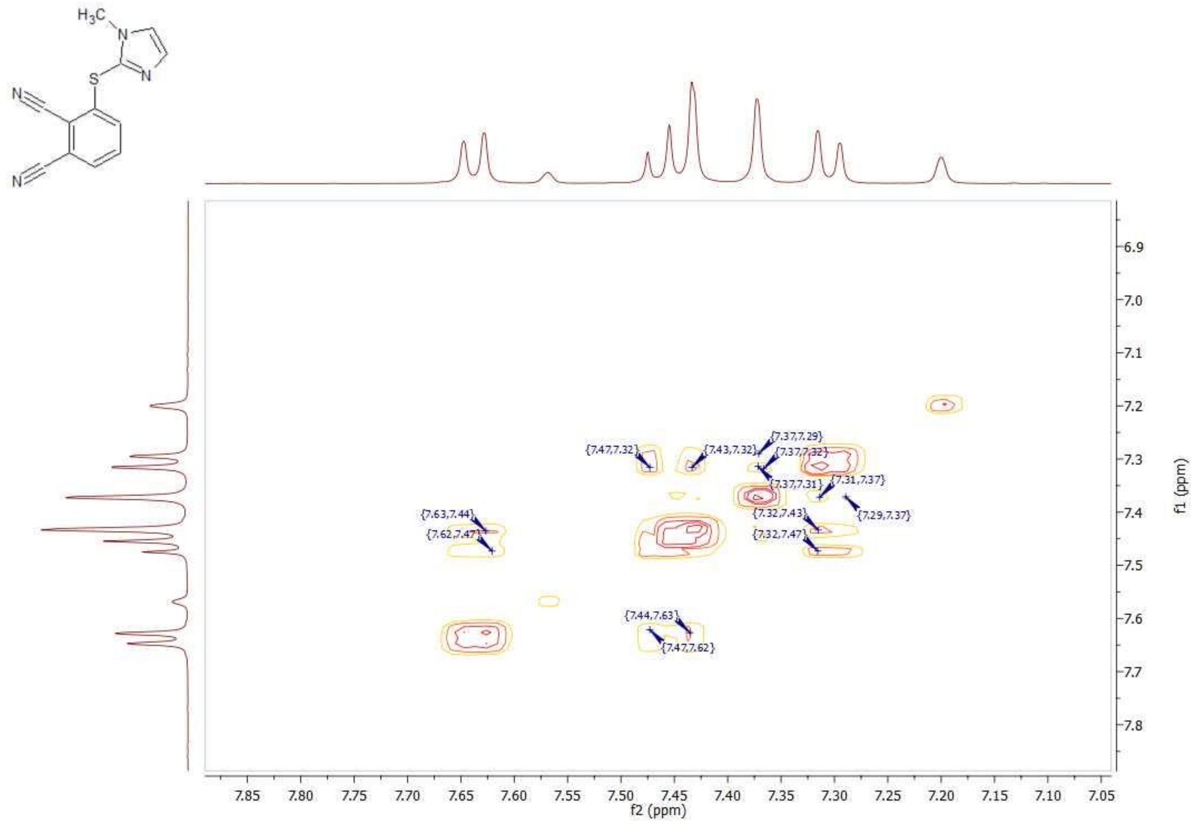

HSQC spectrum of **1** in pyridine-$d_5$

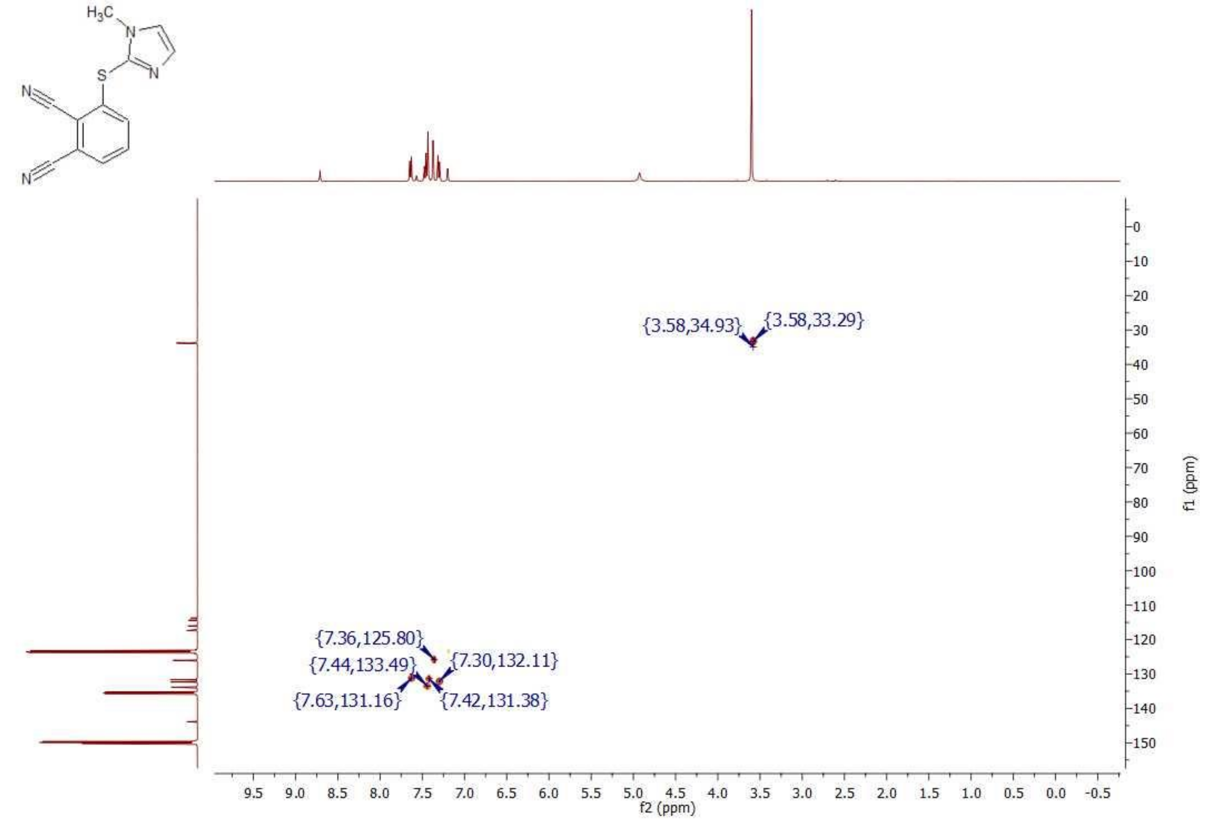


HMBC spectrum of **1** in pyridine-$d_5$

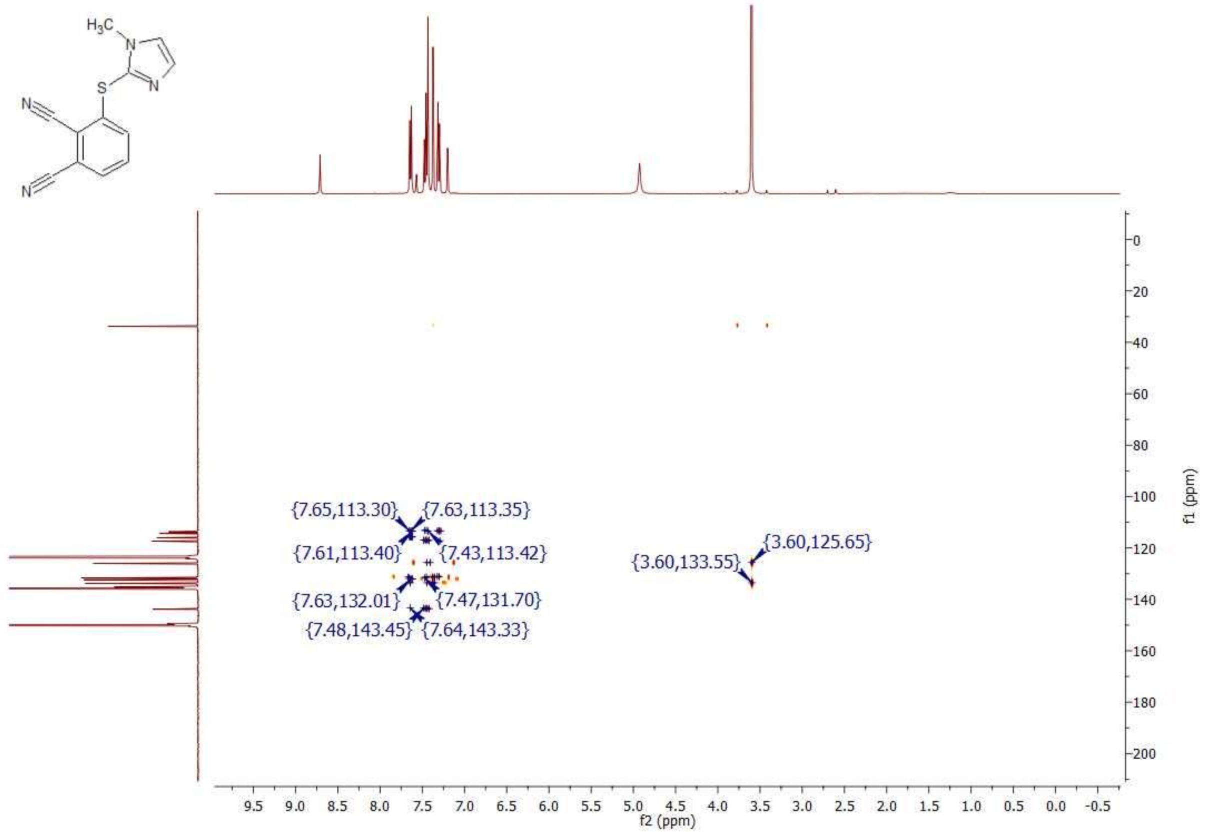

$^{1}H$ NMR spectrum of **2** in DMSO-$d_6$

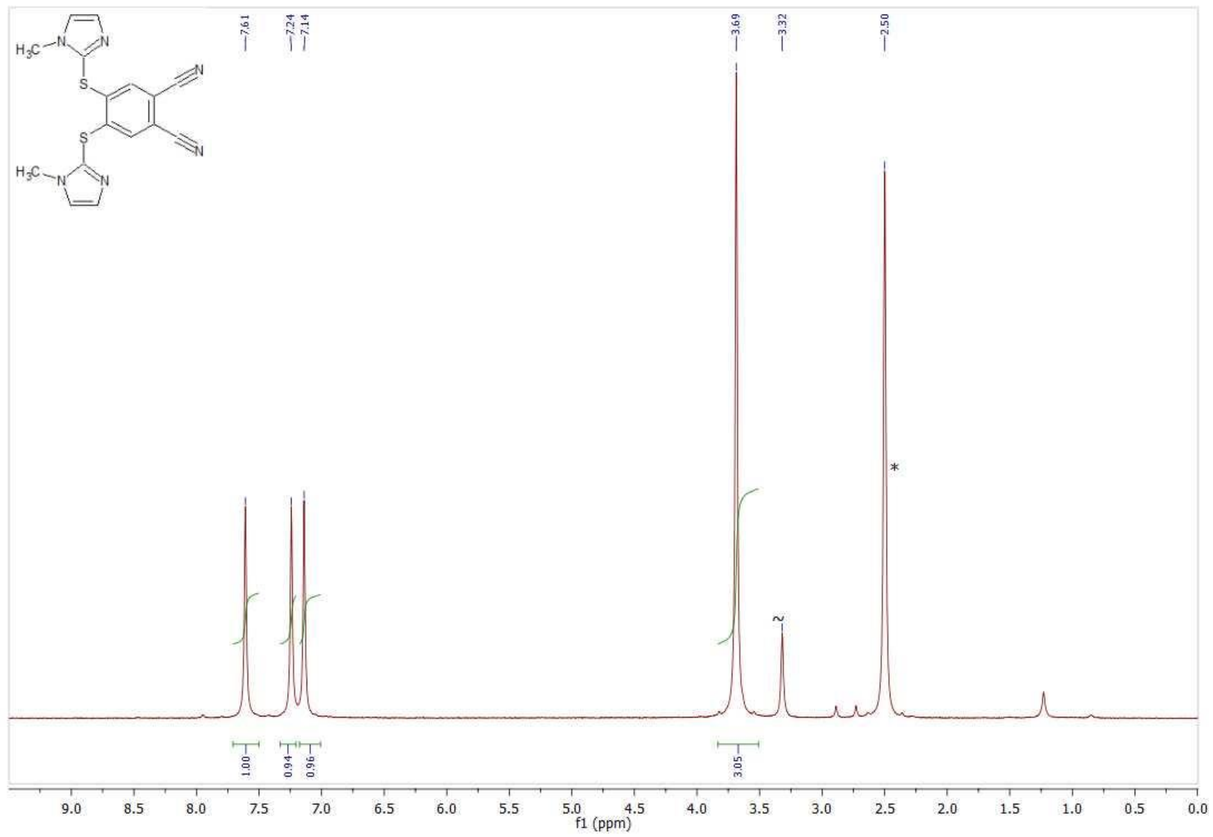


$^{13}C$ NMR spectrum of **2** in DMSO-$d_6$

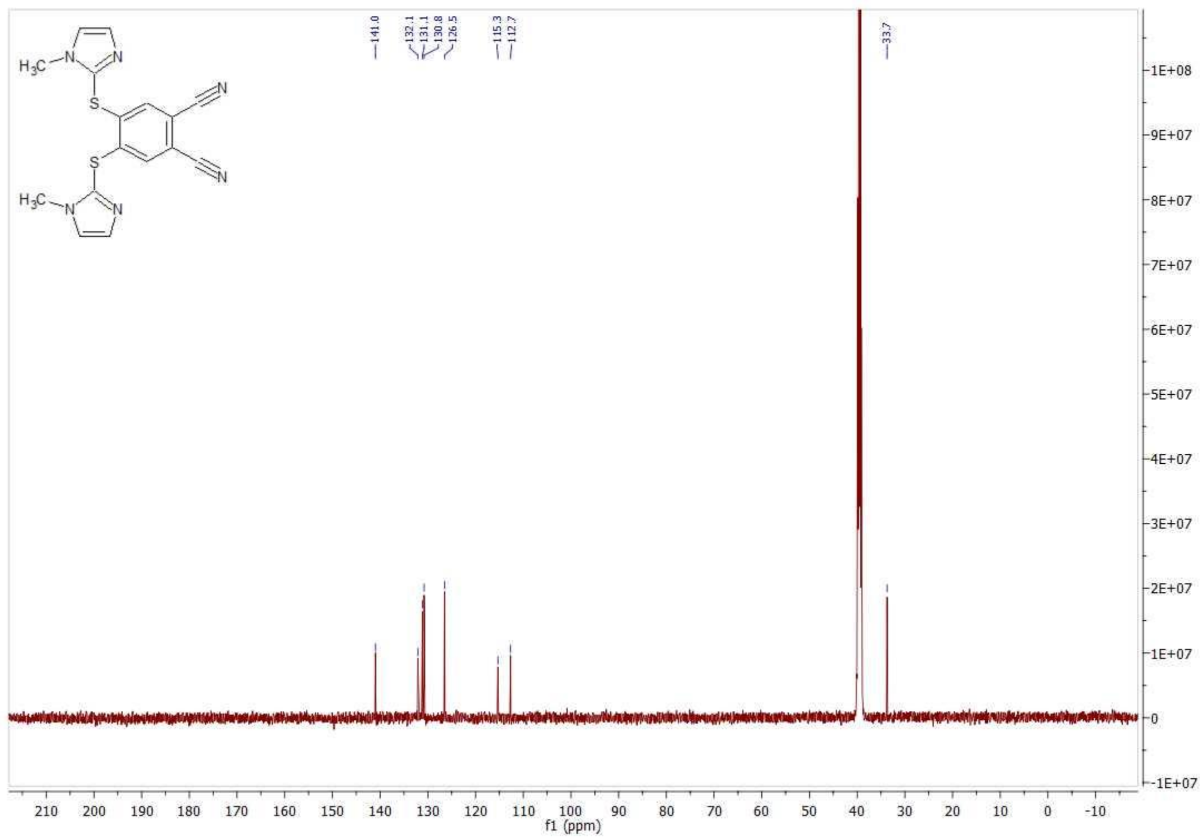

$^{1}H$-$^{1}H$ COSY spectrum of **2** in DMSO-$d_6$

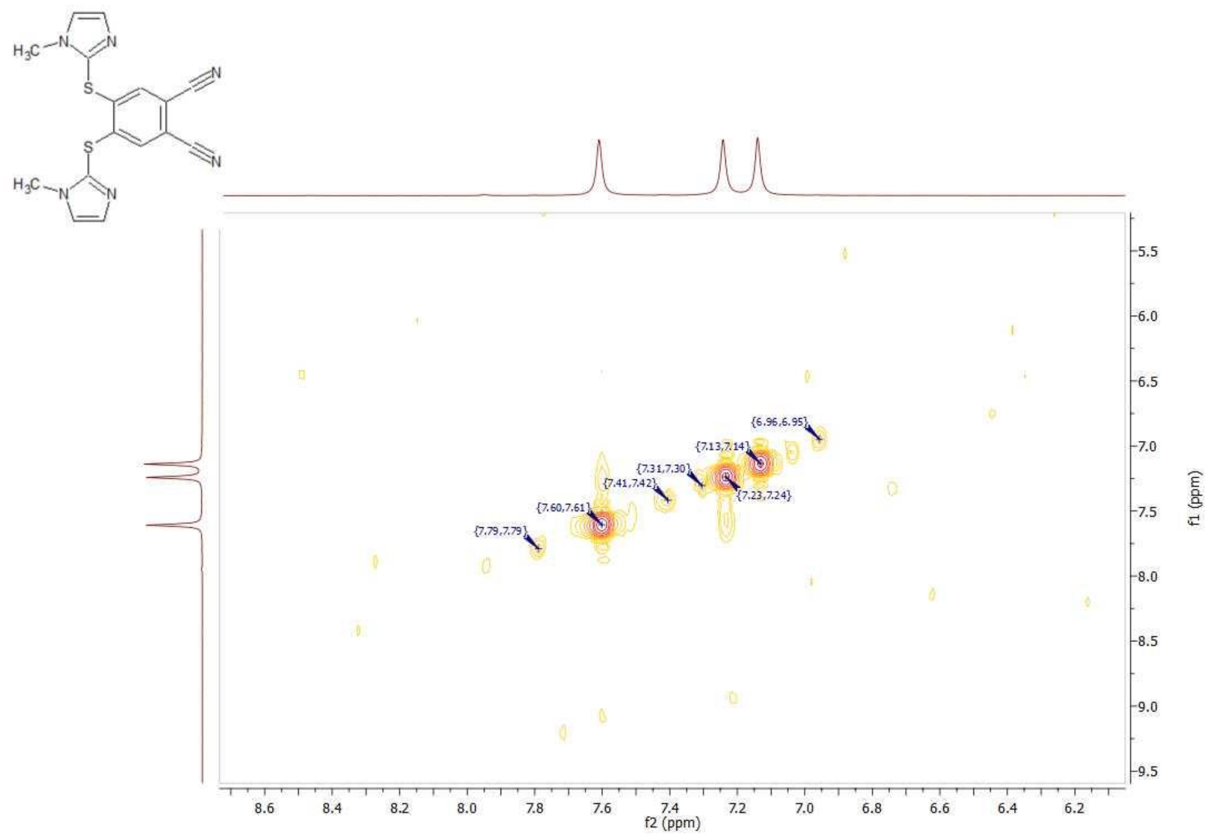


HSQC spectrum of **2** in DMSO-$d_6$

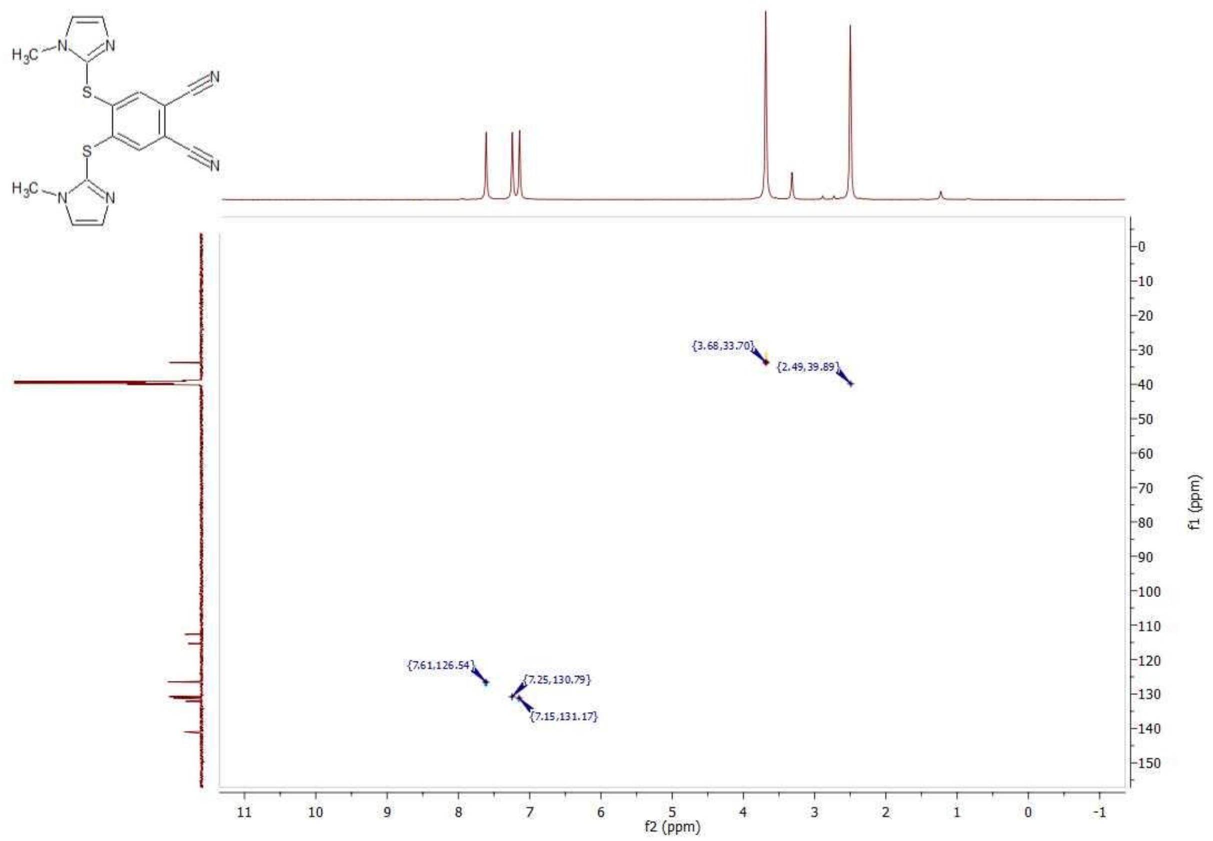

HMBC spectrum of **2** in DMSO-$d_6$

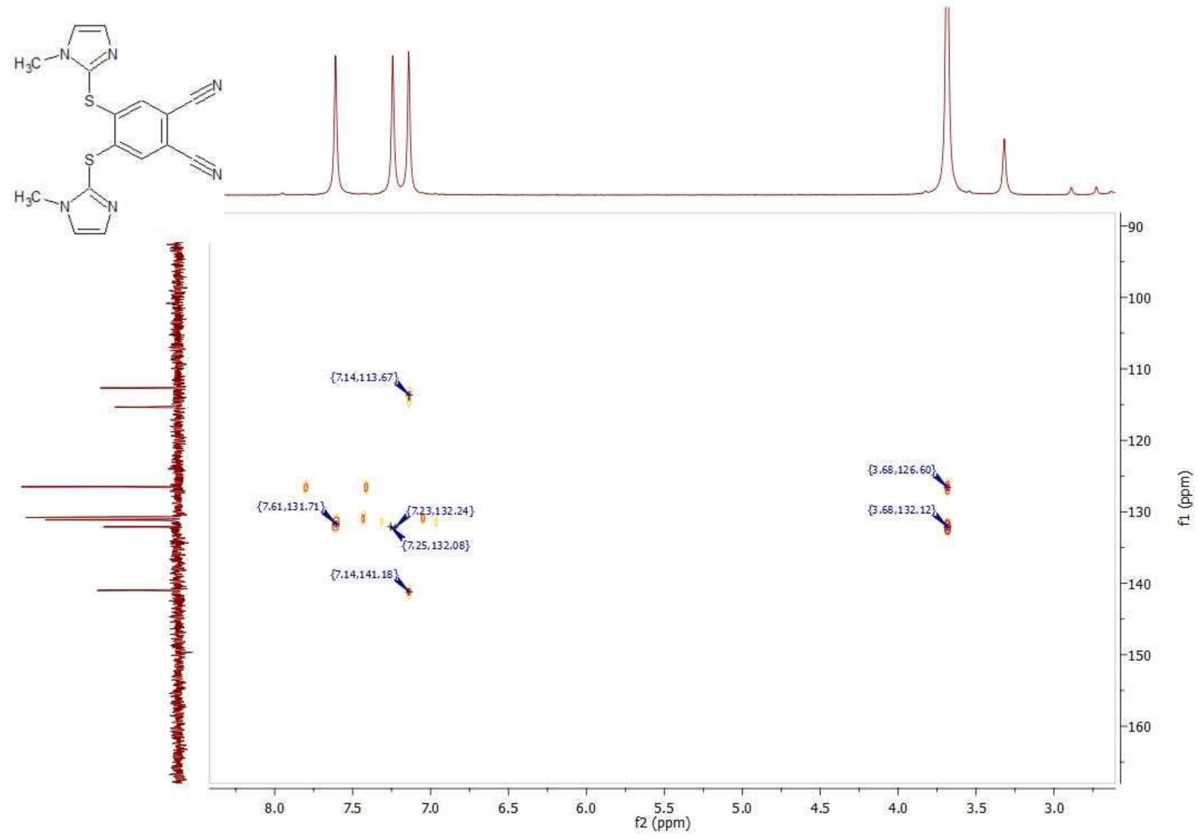


$^{1}H$ NMR spectrum of **3** in DMSO-$d_6$

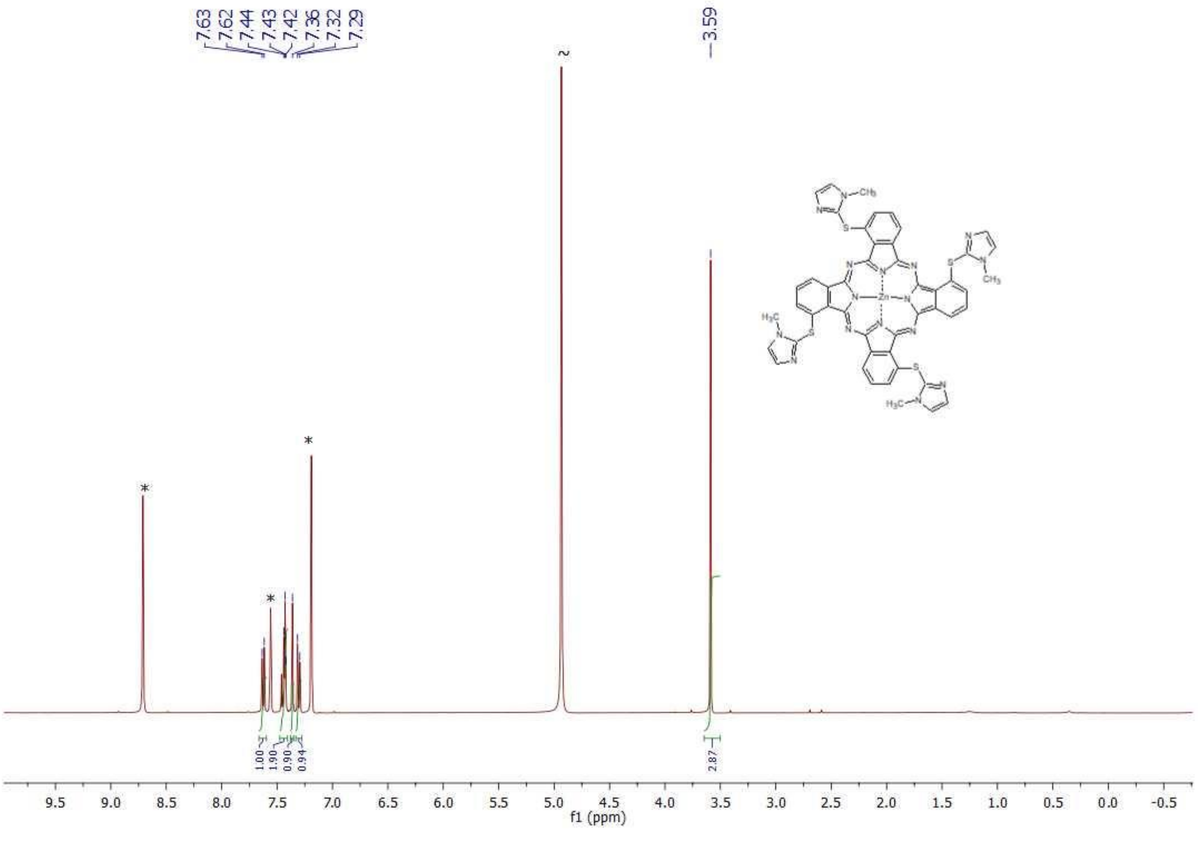

$^{13}C$ NMR spectrum of **3** in DMSO-$d_6$

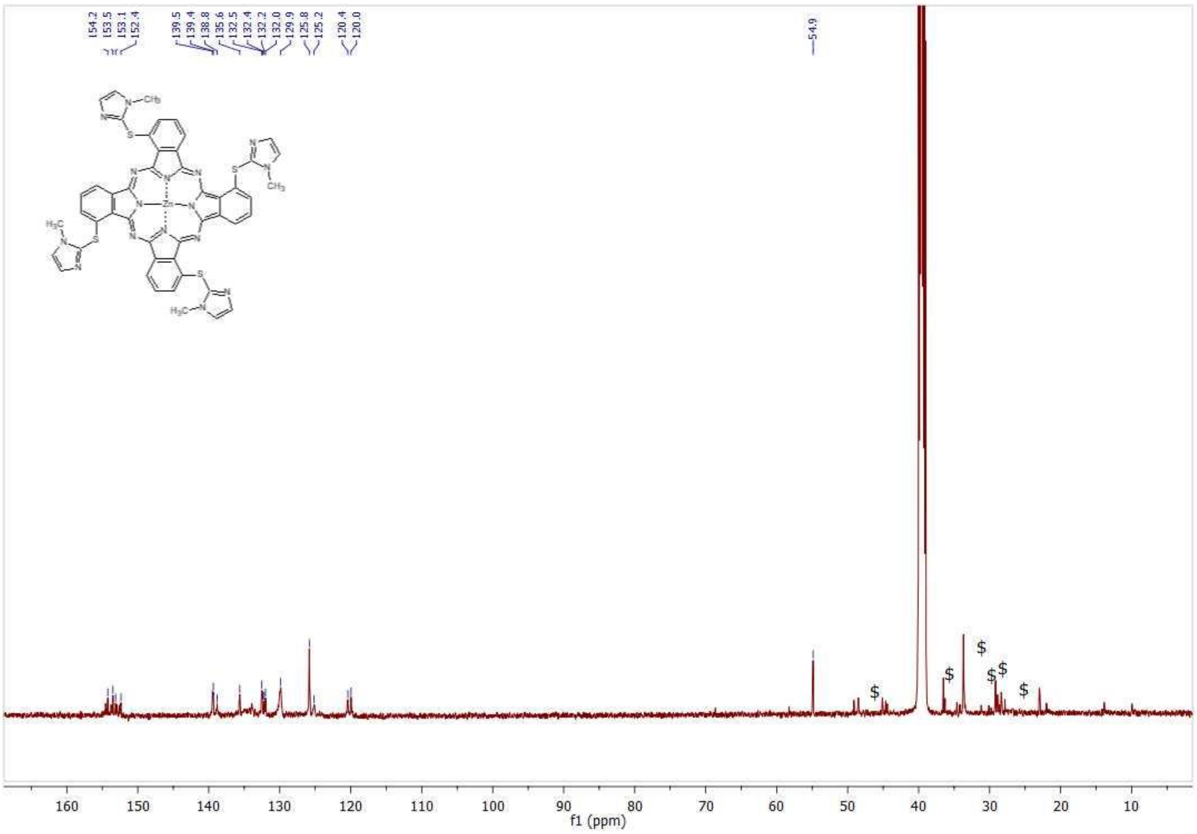


$^{1}H$ NMR spectrum of **6** in DMSO-$d_6$

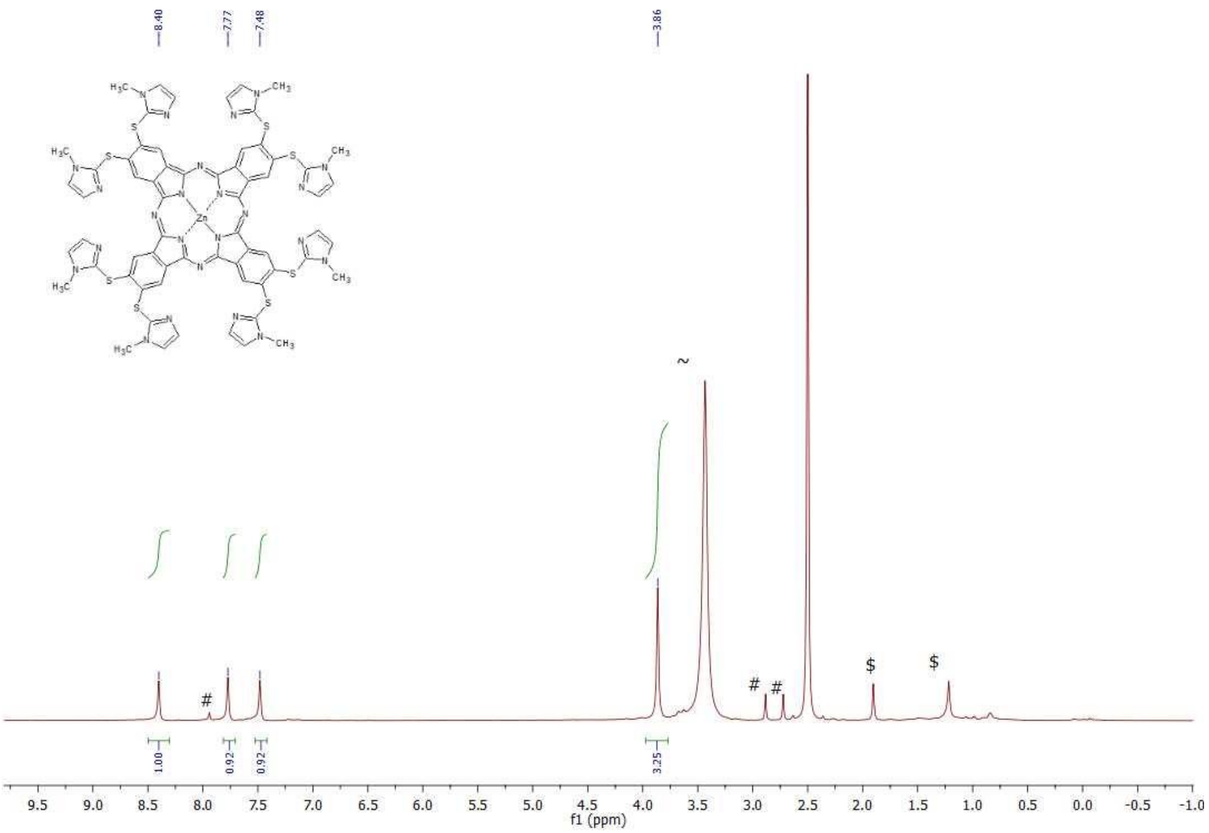

$^{13}C$ NMR spectrum of **6** in DMSO-$d_6$

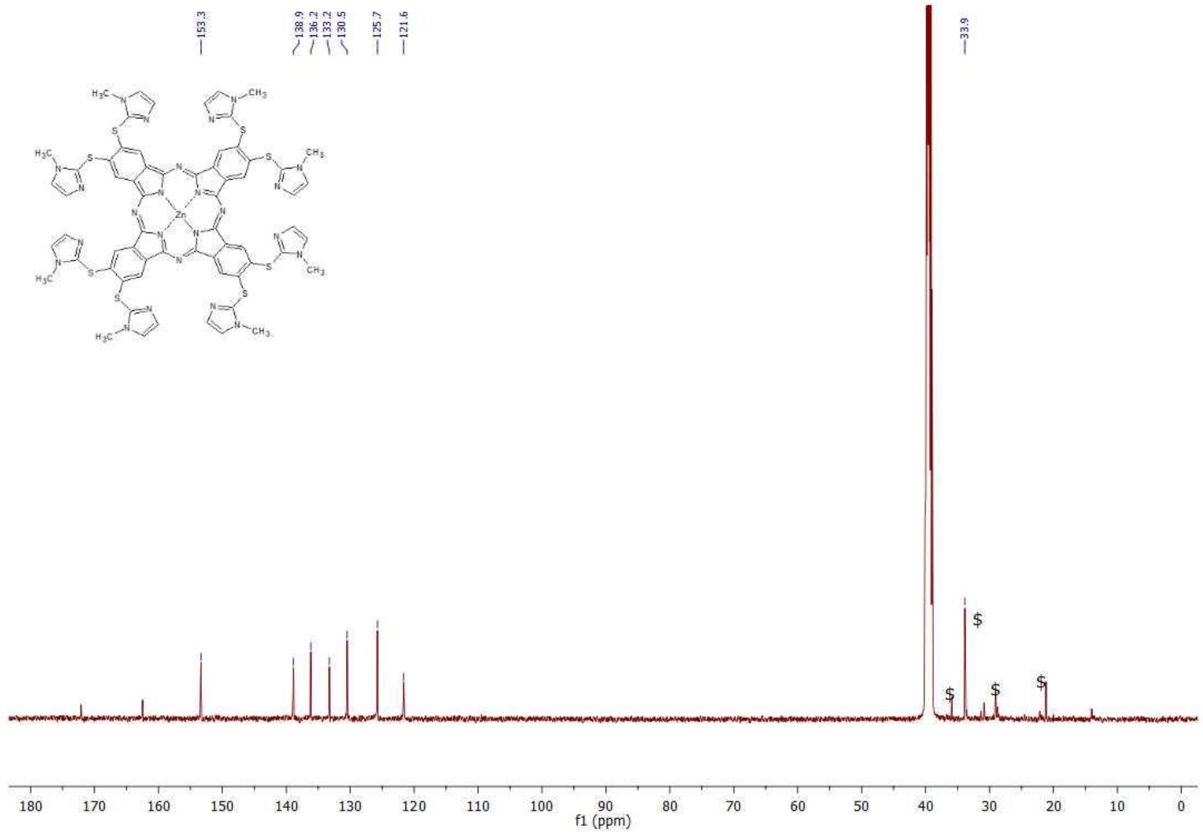


$^{1}H$ NMR spectrum of **7** in DMSO-$d_6$

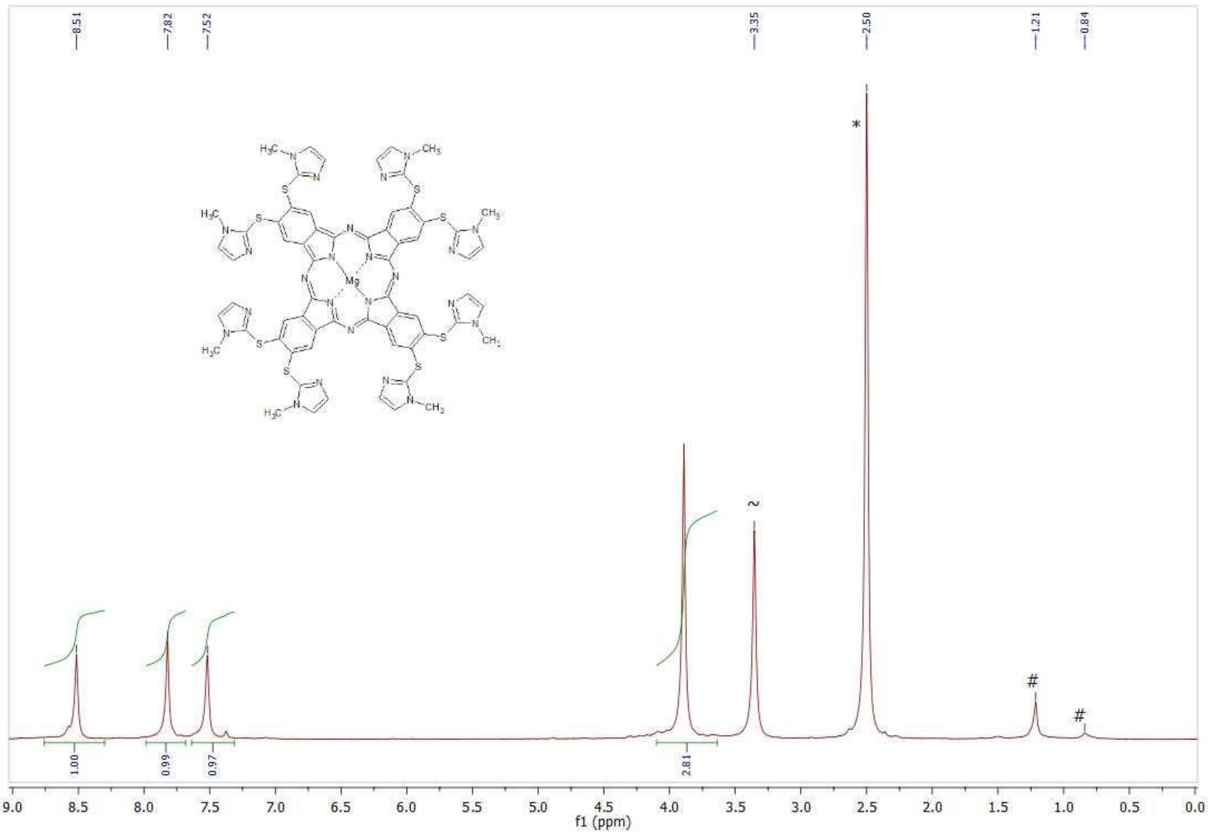


$^{13}C$ NMR spectrum of **7** in pyridine-$d_5$

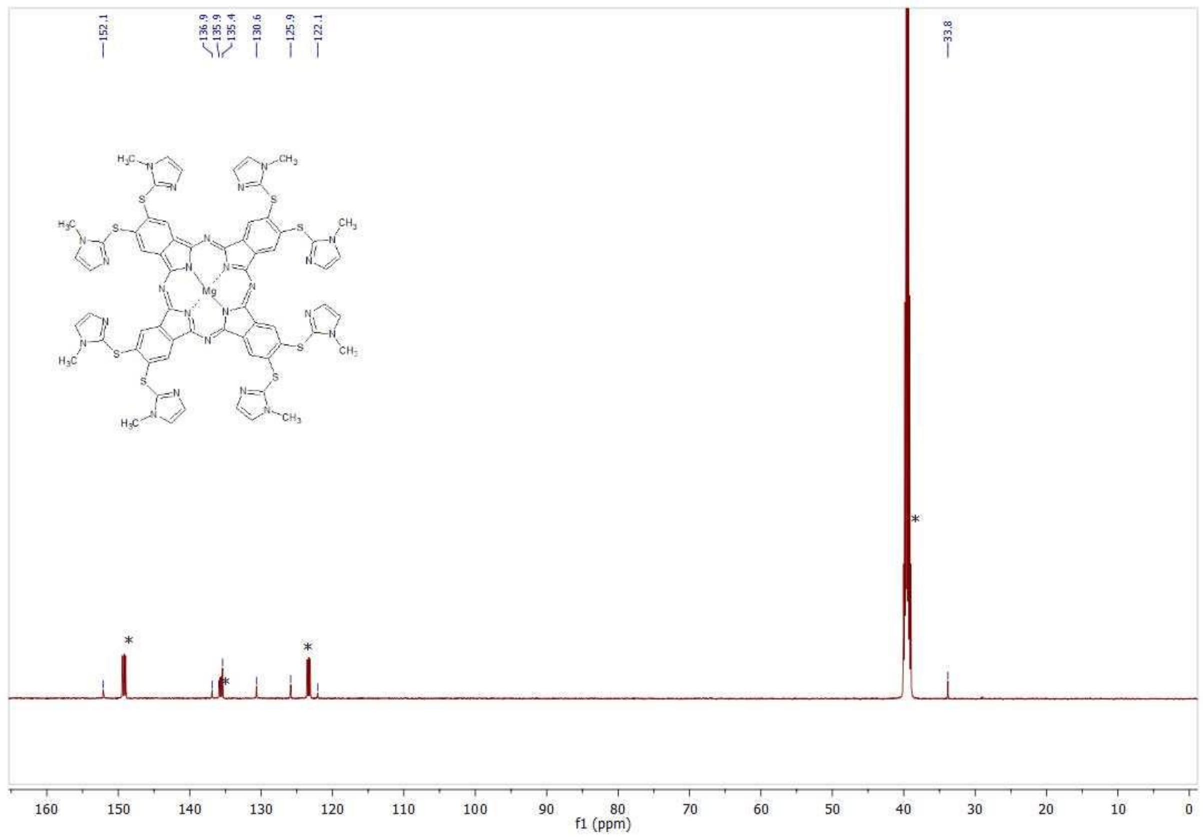


$^{1}H$-$^{1}H$ COSY spectrum of **7** in DMSO-$d_6$

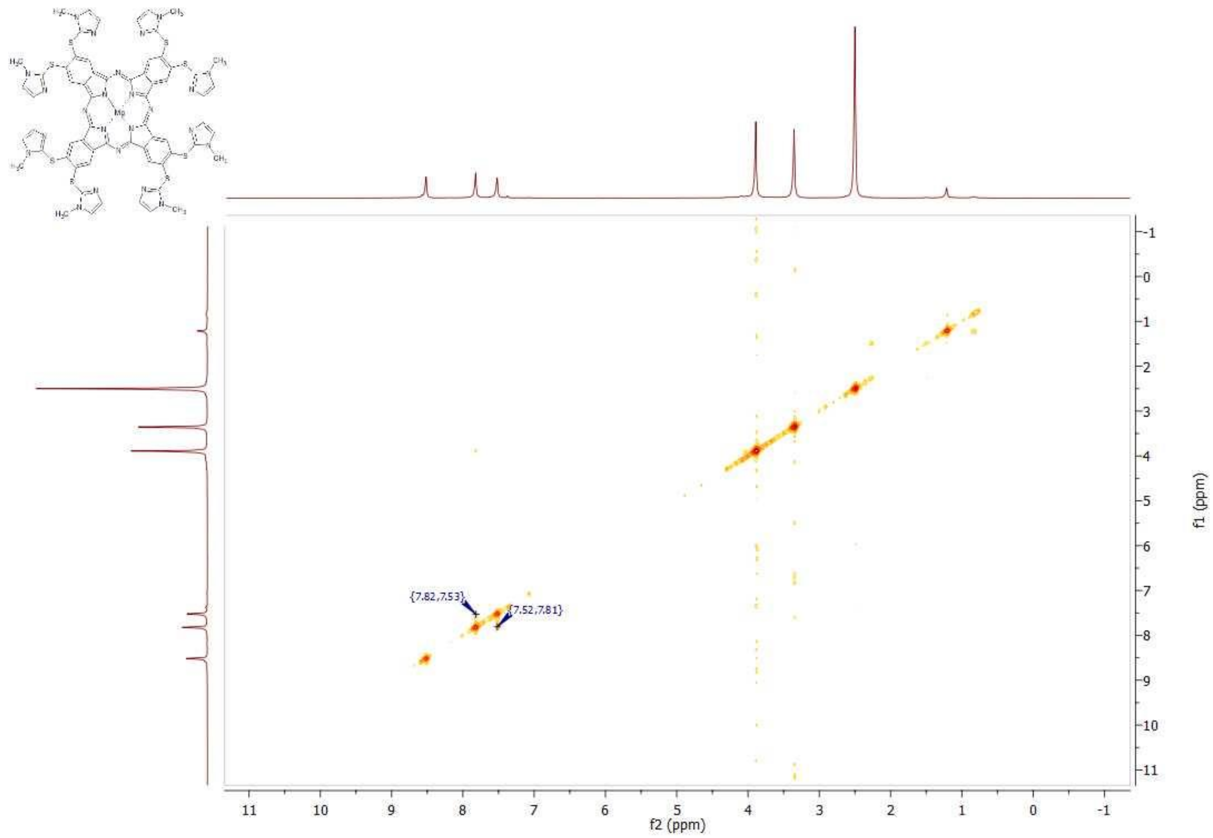


HSQC spectrum of **7** in DMSO-$d_6$

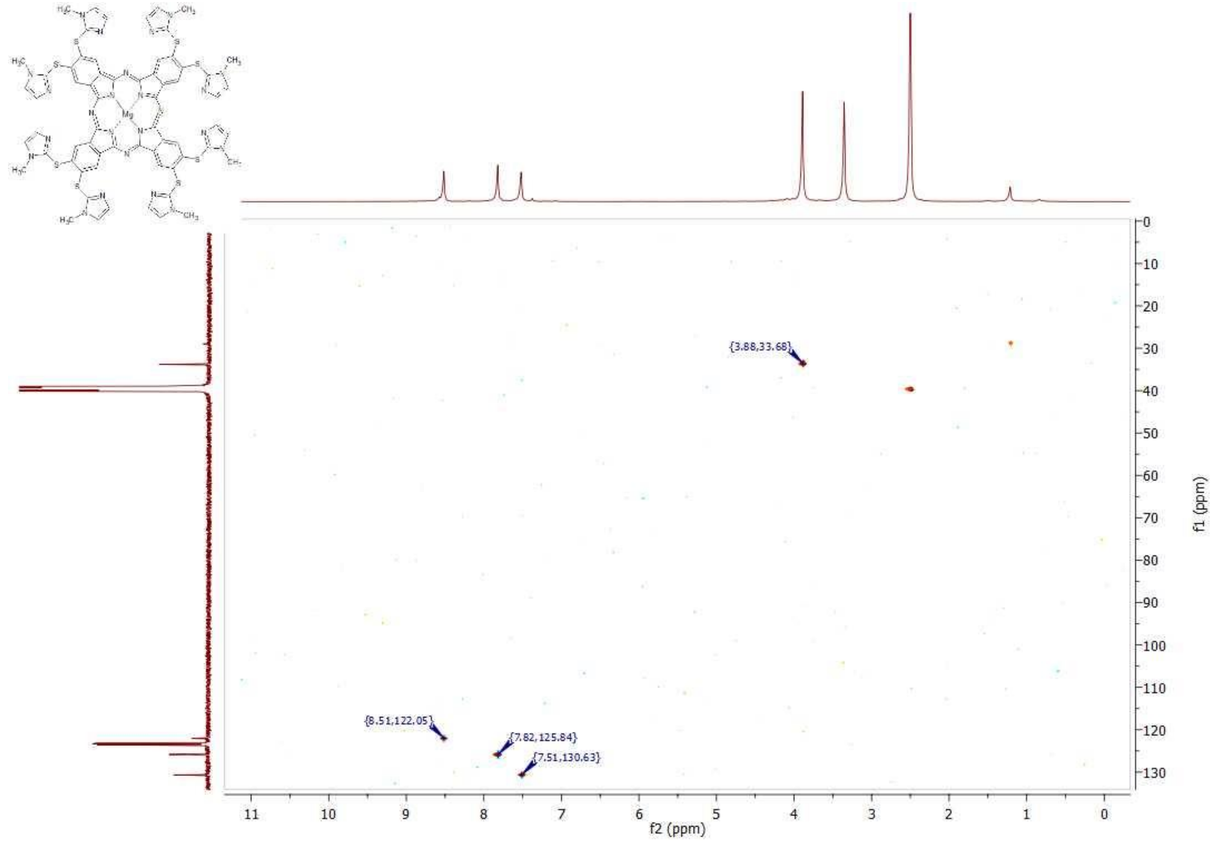


HMBC spectrum of **7** in DMSO-$d_6$

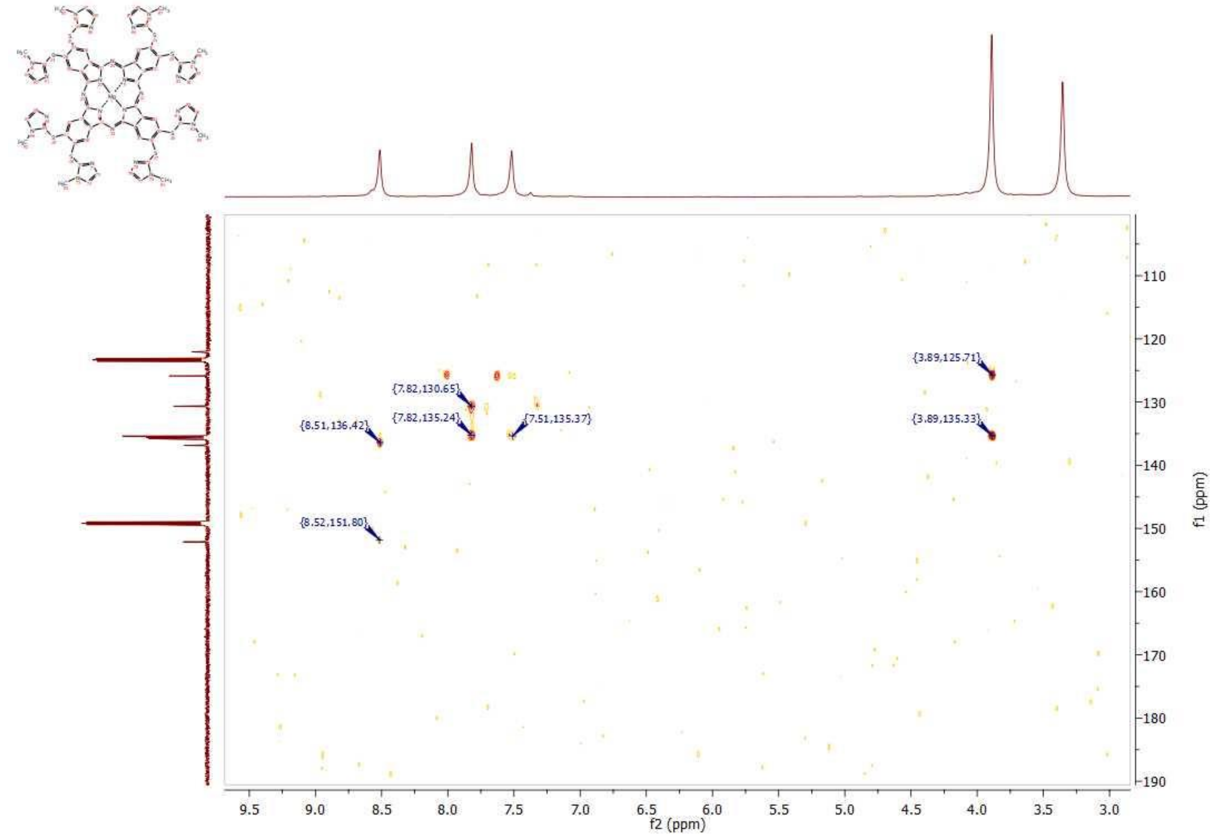

2. Mass spectra data

ESI spectra of compound **1**.

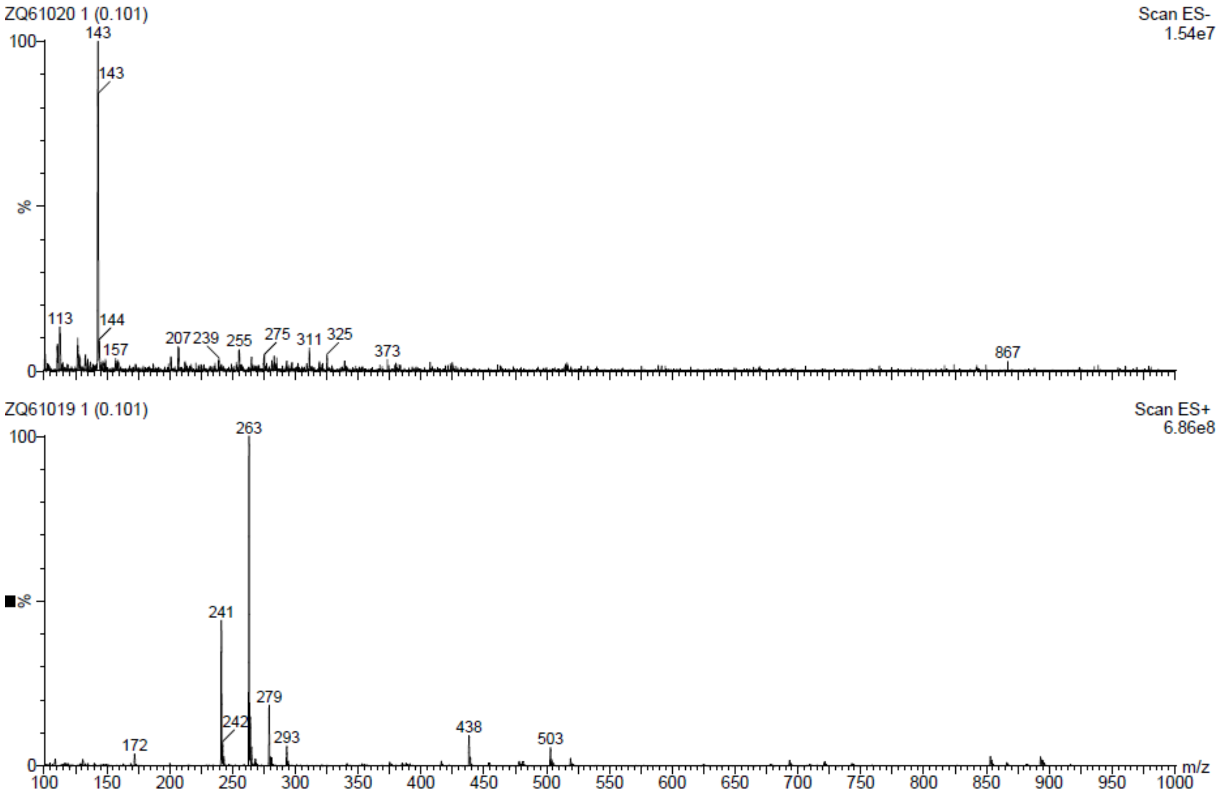


ESI spectra of compound **2**

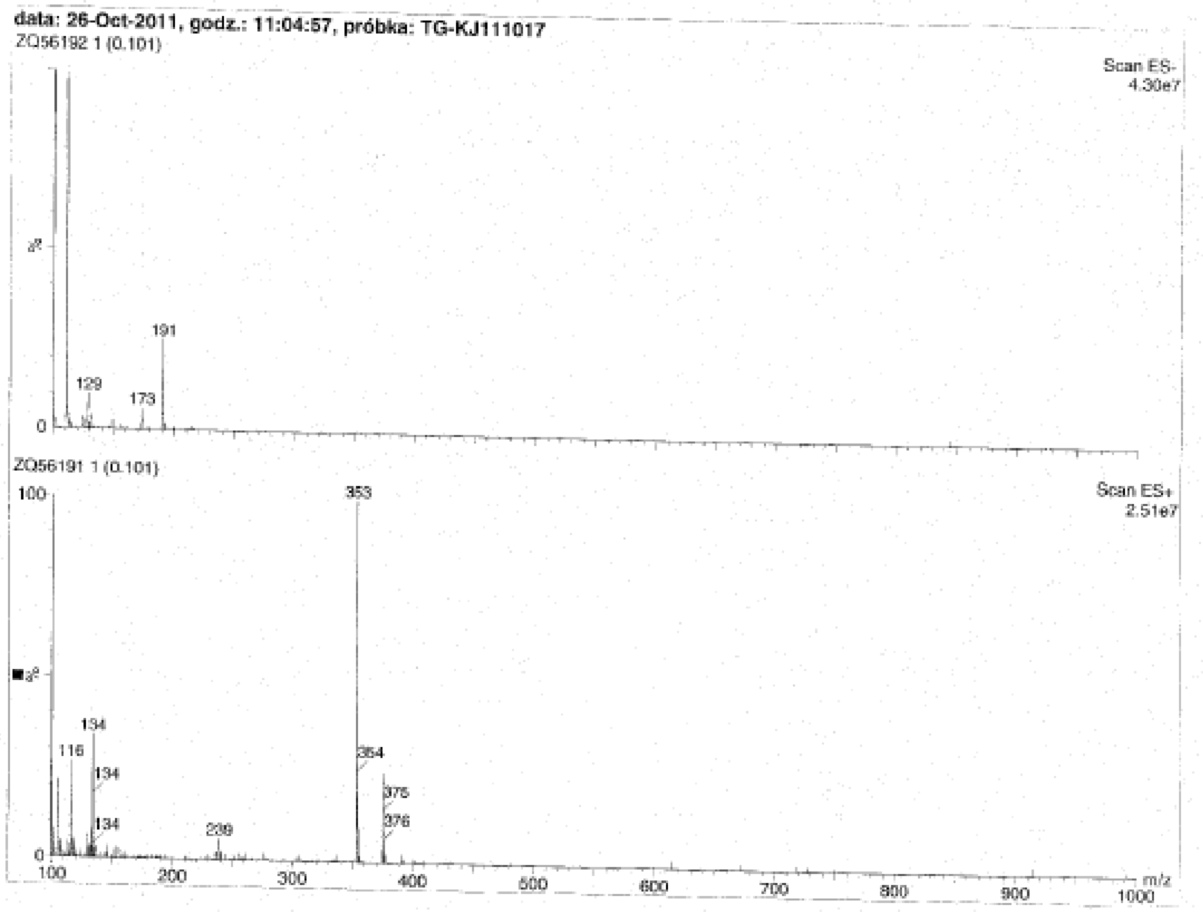

Maldi-TOF spectra of compound **3**

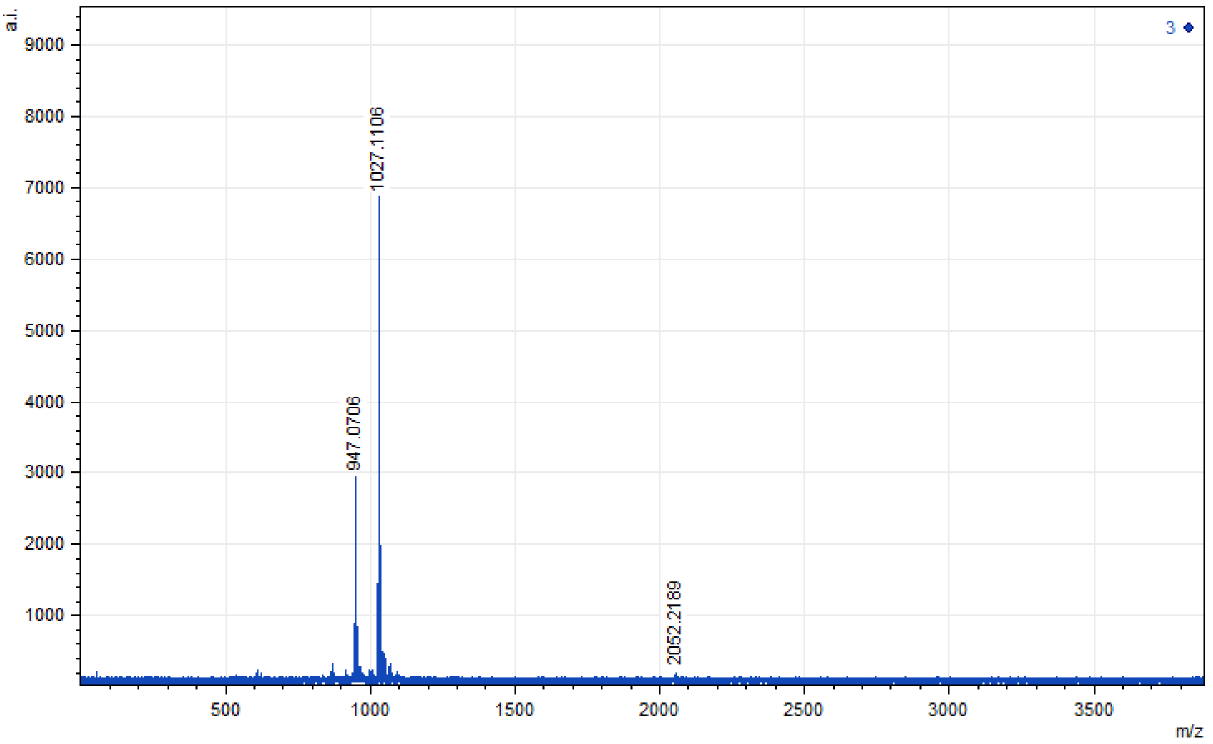


Maldi-TOF/TOF Lift spectra of compound **3**

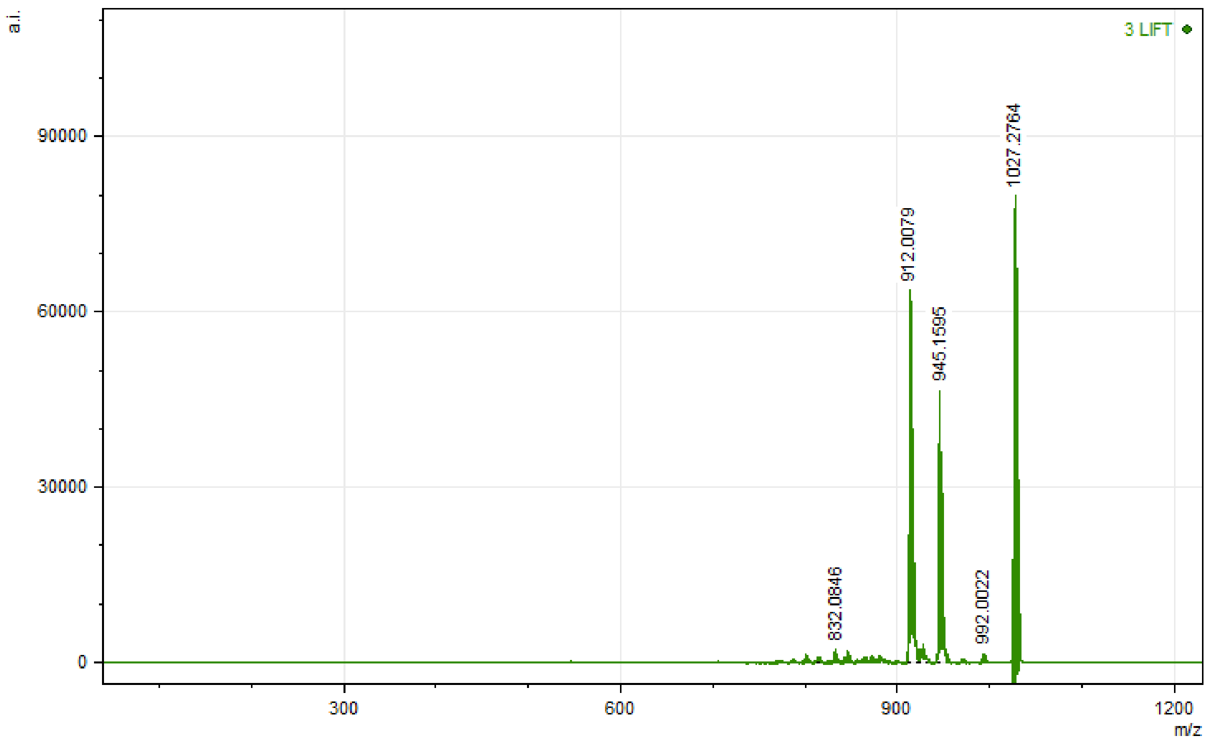

Maldi-TOF spectra of compound **4**

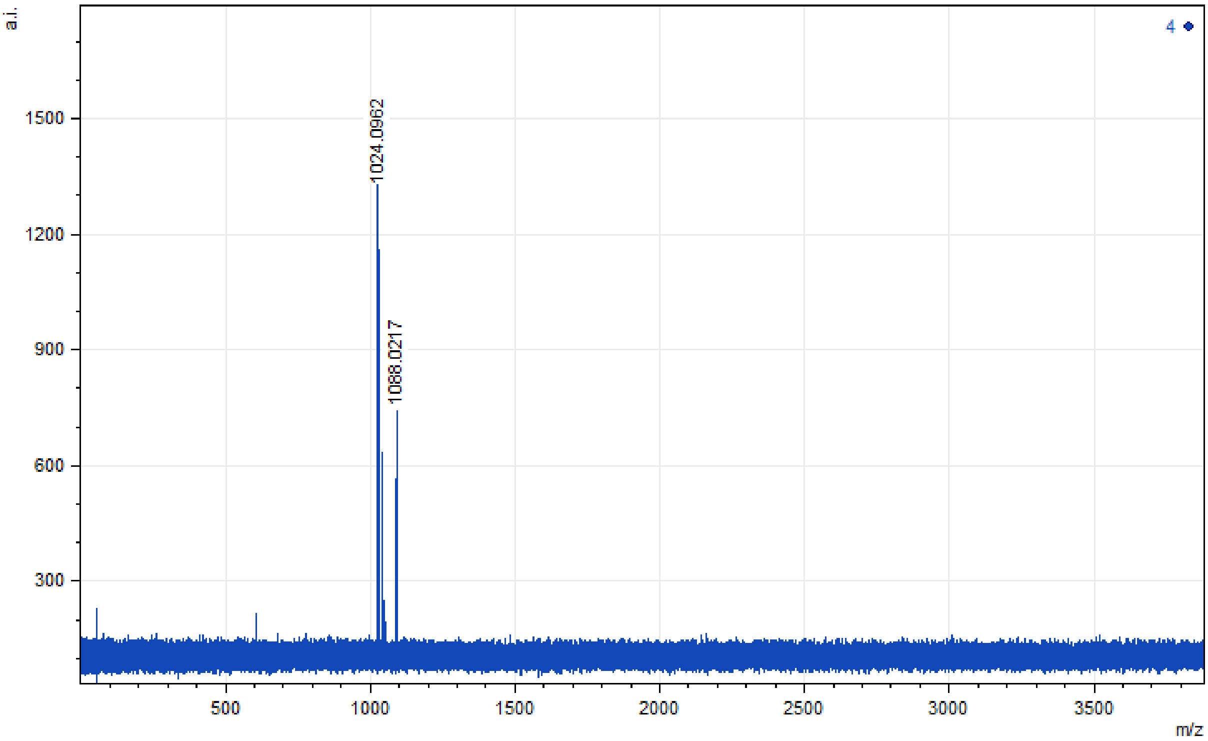


Maldi-TOF/TOF Lift spectra of compound **4**

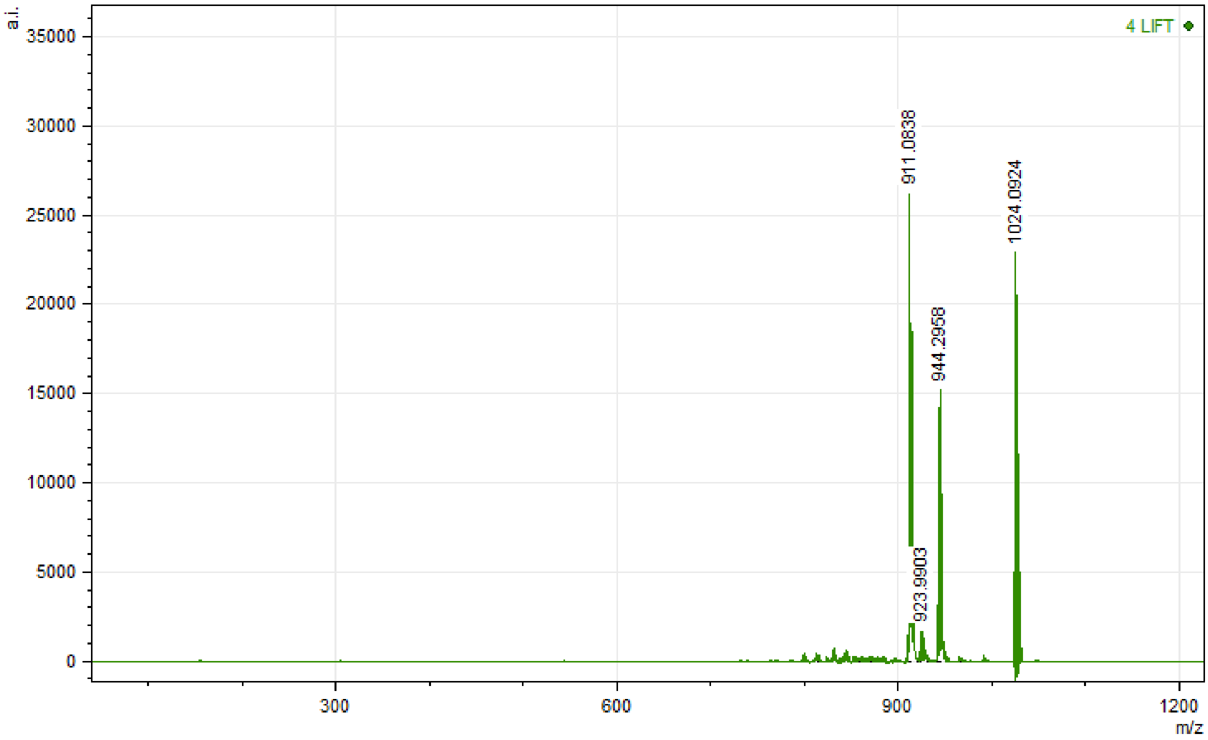

Maldi-TOF spectra of compound **5**

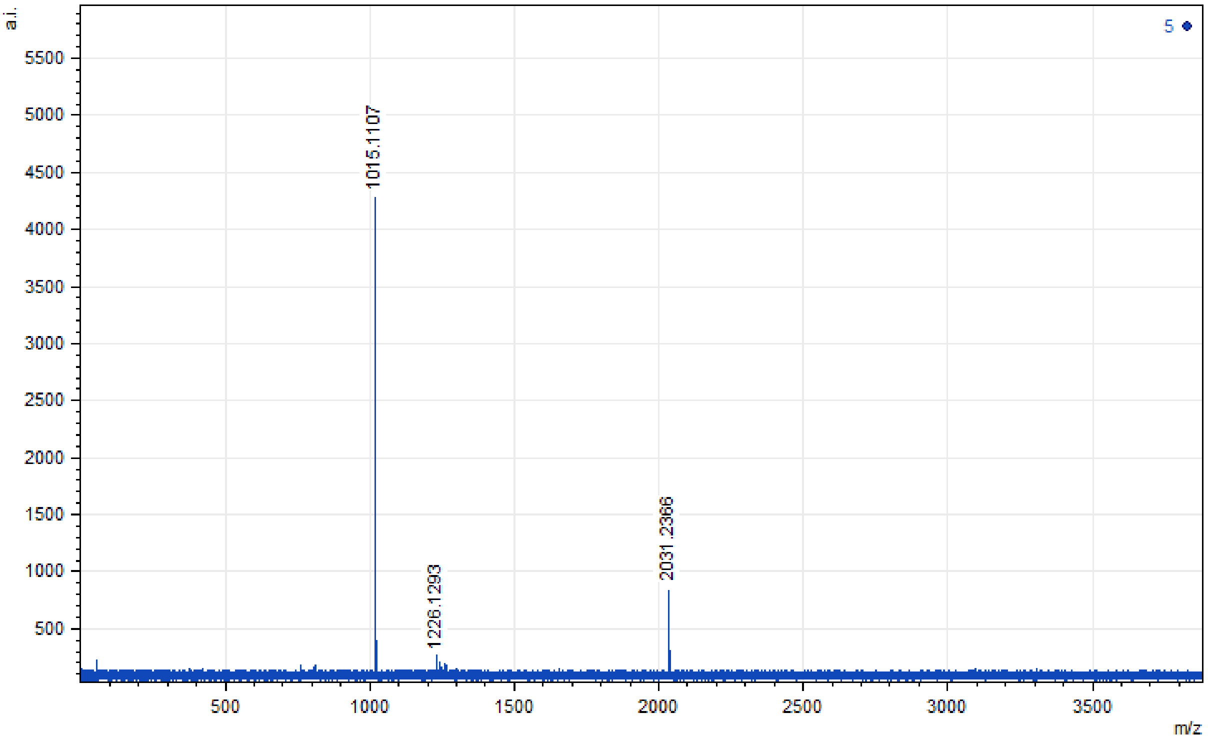


Maldi-TOF/TOF Lift spectra of compound **5**

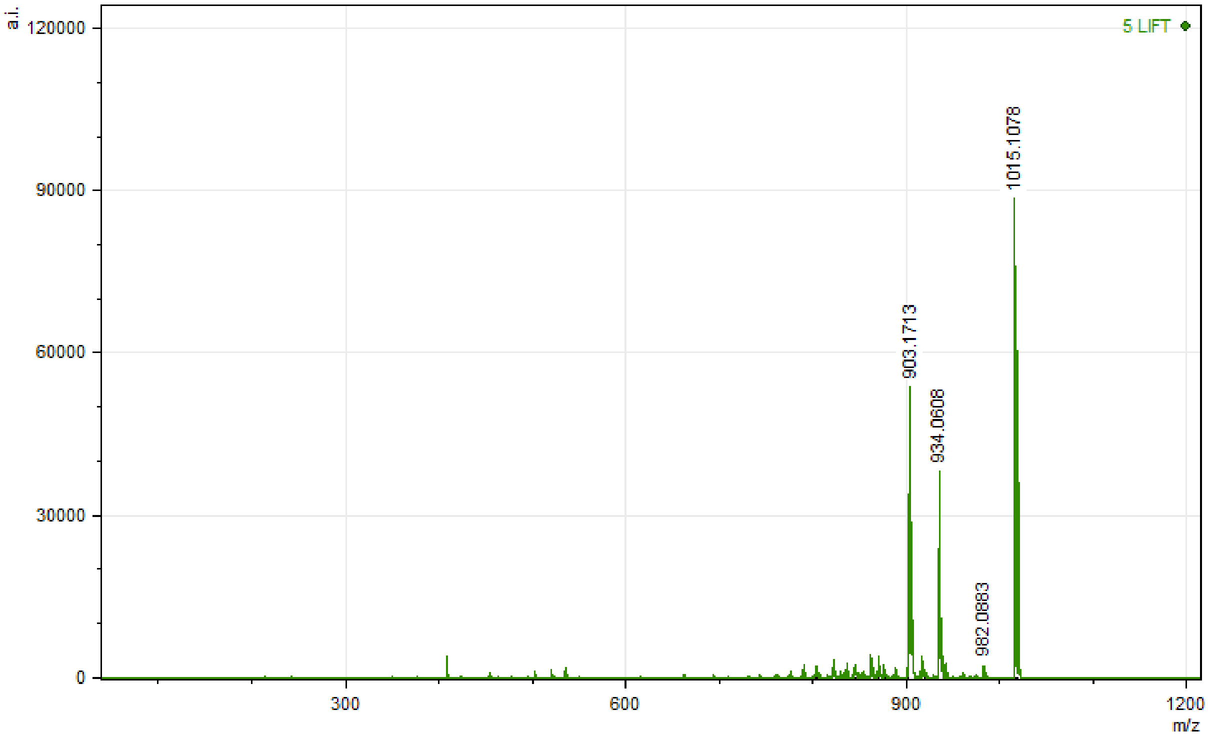

Maldi-TOF spectra of compound **6**

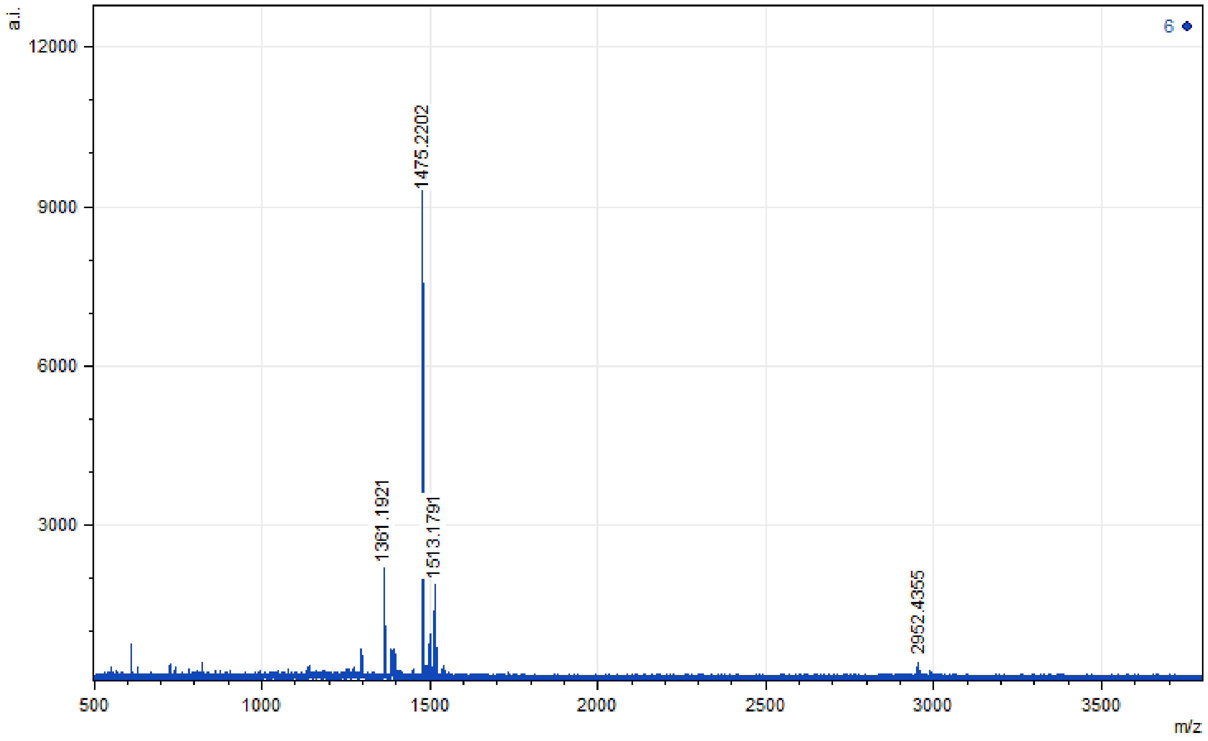


Maldi-TOF/TOF Lift spectra of compound **6**

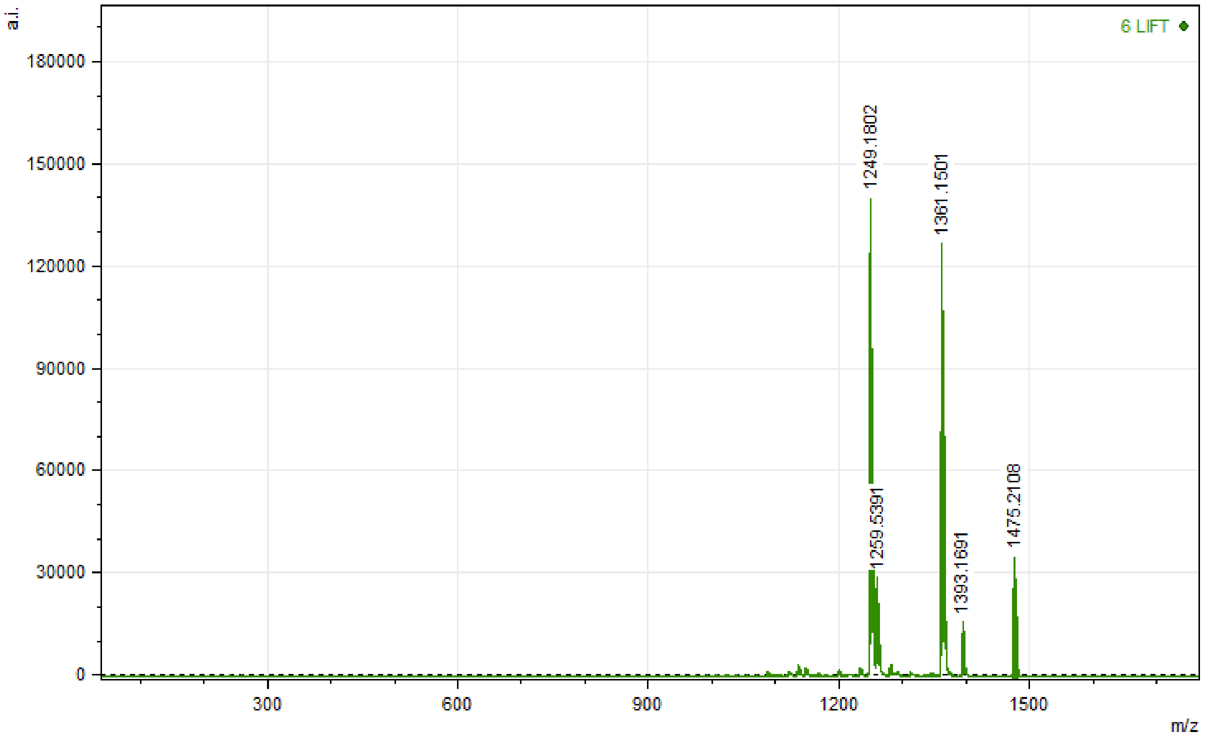

Maldi-TOF spectra of compound **7**

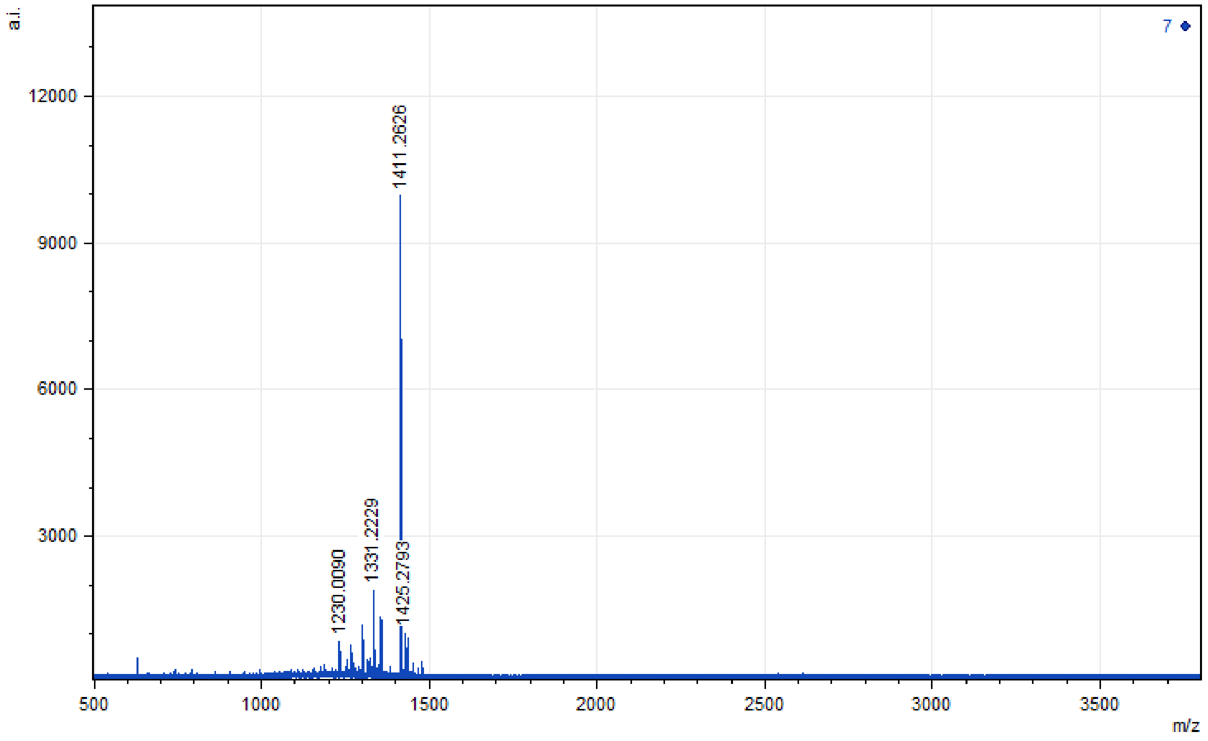


Maldi-TOF/TOF Lift spectra of compound **7**

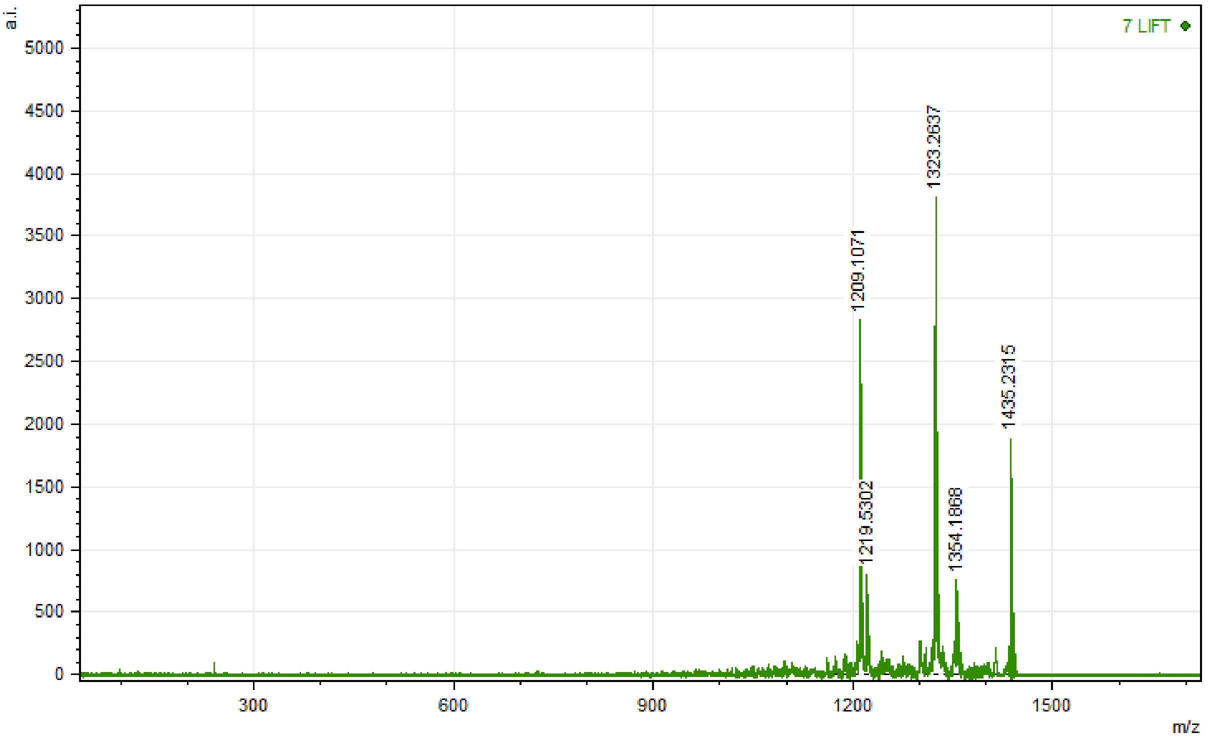

3. IR data

IR spectra of compound **3**

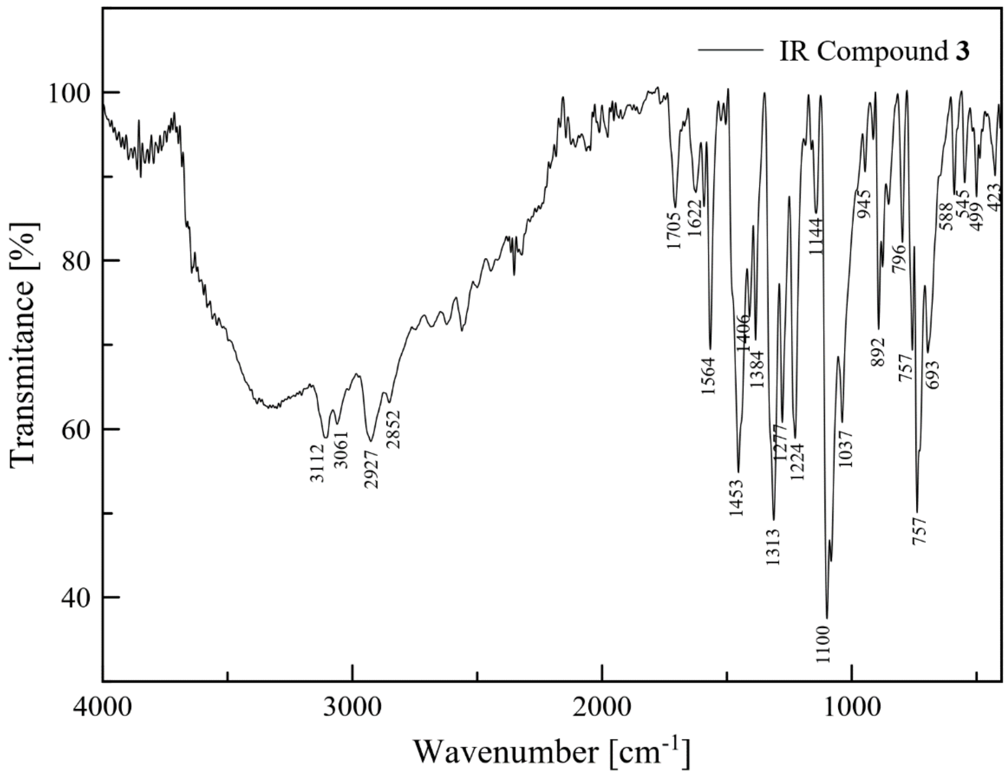


IR spectra of compound **4**

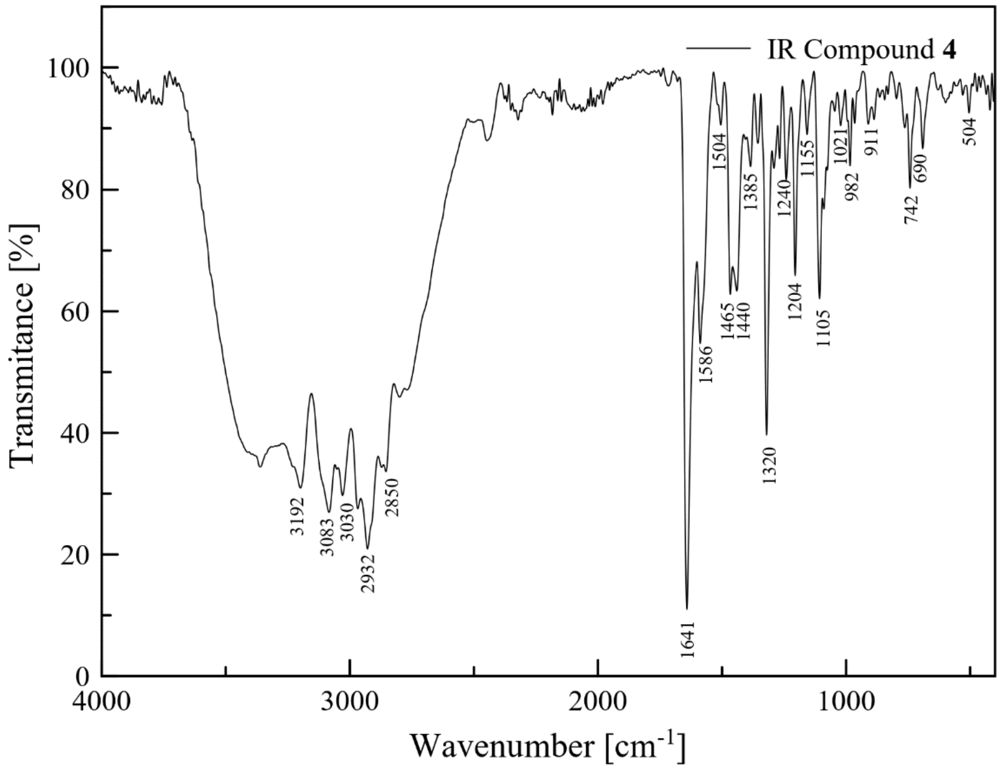

IR spectra of compound **5**

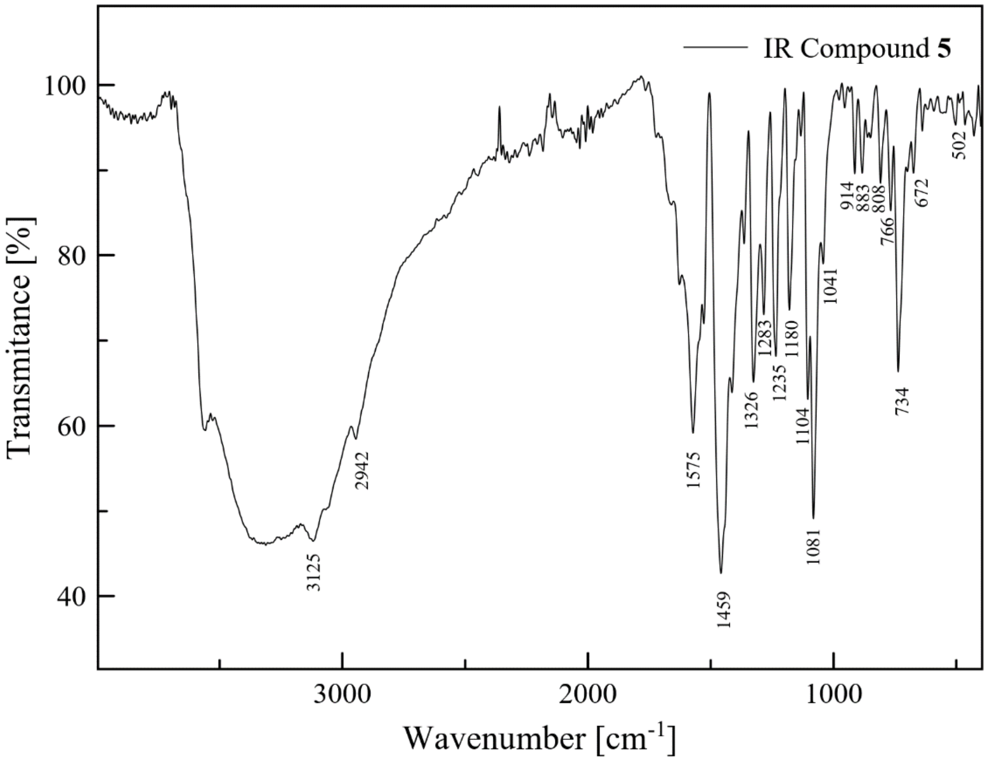


IR spectra of compound **6**

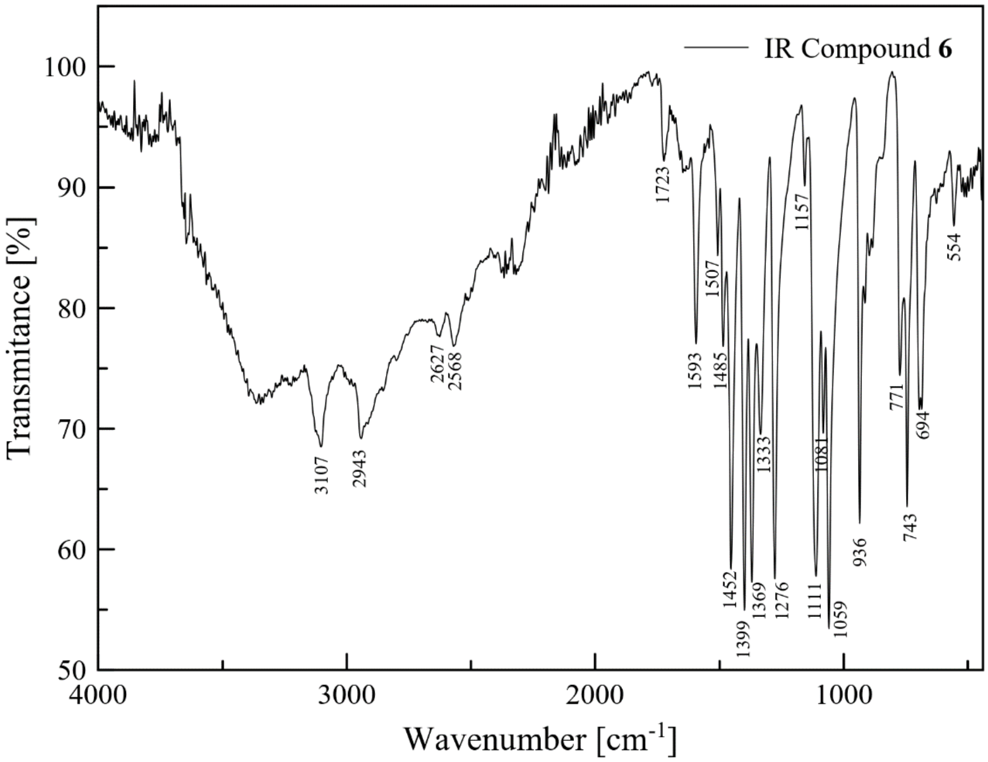

IR spectra of compound **7**

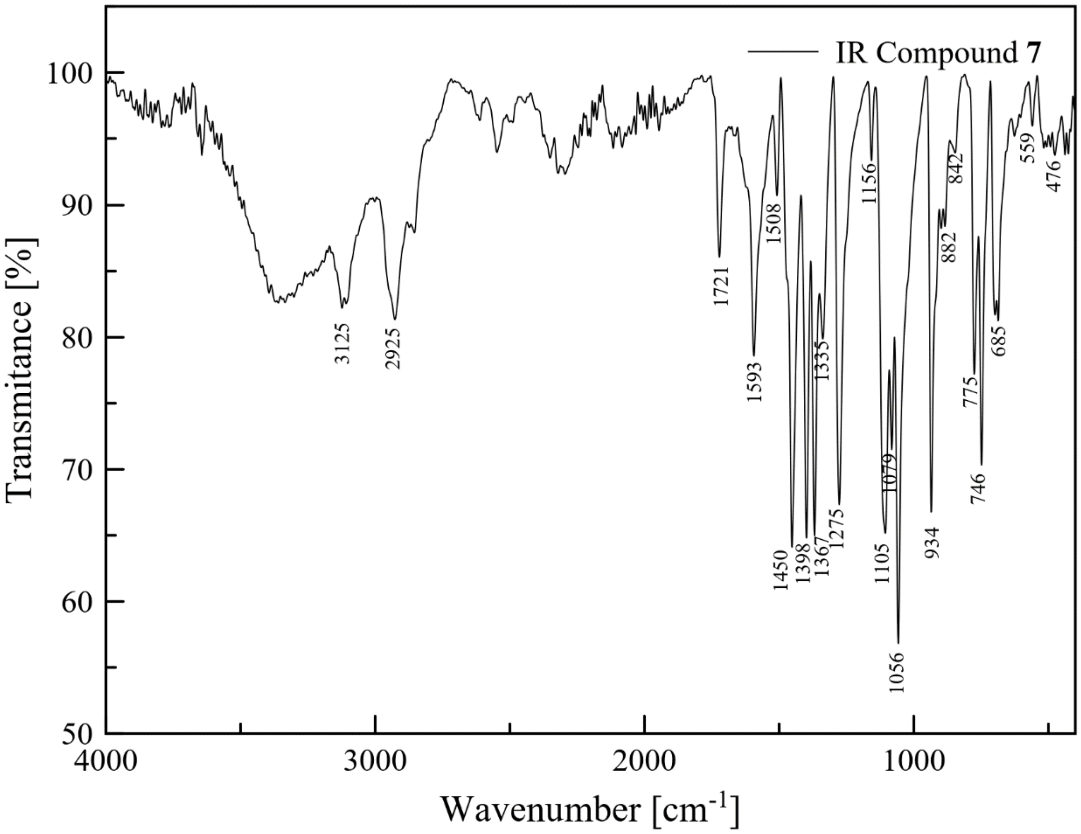

4. HPLC data

Compound **3**

Analytical HPLC was carried out on an Agilent 1200 instrument equipped with an UV-DAD detector. The chromatographic separation was achieved on an octadecylsilane coated columns, 150 mm × 4.6 mm, 5 µm (Eclipse XDB-C18, Agilent), using isocratic and linear gradient conditions at a flow rate of 1.0 mL/min in three different phases configurations.

Phases configuration 1

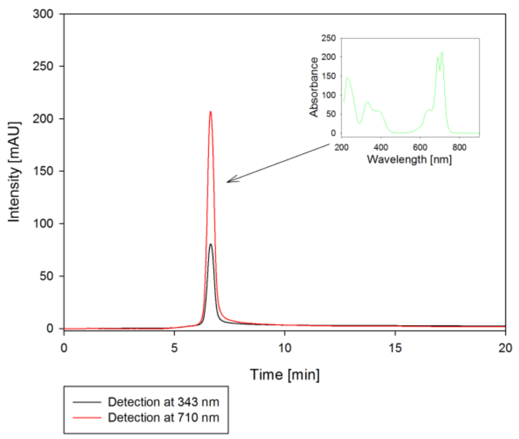


Phase

| Time | MeOH | H2O |
|---|---|---|
| 0 | 85 | 15 |
| 2 | 85 | 15 |
| 3 | 99 | 1 |
| 20 | 99 | 1 |

| Detection at 343 nm | | | | Detection at 710 nm | | | |
|---|---|---|---|---|---|---|---|
| Signal | Retention time [min] | Area | Content [%] | Signal | Retention time [min] | Area | Content [%] |
| 1 | 6,64 | 1765,1 | 100,00 | 1 | 6,64 | 4711,8 | 100,00 |

Phases configuration 2

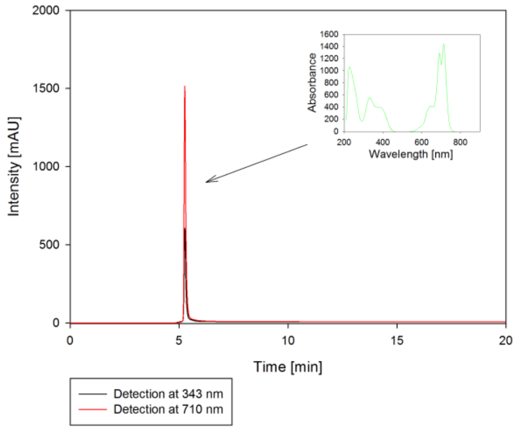


Phase

| Time | MeOH | H2O | CH2Cl2 |
|---|---|---|---|
| 0 | 80 | 15 | 5 |
| 2 | 80 | 15 | 5 |
| 3 | 60 | 1 | 39 |
| 20 | 60 | 1 | 39 |

Detection at 343 nm

| Signal | Retention time [min] | Area | Content [%] |
|---|---|---|---|
| 1 | 5,26 | 3800,8 | 100,00 |

Detection at 710 nm

| Signal | Retention time [min] | Area | Content [%] |
|---|---|---|---|
| 1 | 5,26 | 8975,2 | 100,00 |

## Phases configuration 3

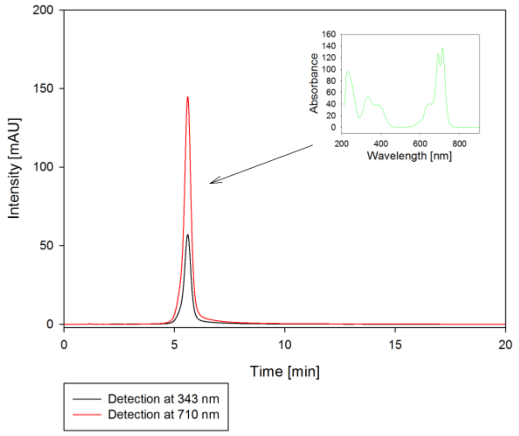


Phase

| MeOH | H2O | CH2Cl2 |
|---|---|---|

| | | |
|---|---|---|
| 70 | 15 | 15 |

| Detection at 343 nm | | | | Detection at 710 nm | | | |
|---|---|---|---|---|---|---|---|
| Signal | Retention time [min] | Area | Content [%] | Signal | Retention time [min] | Area | Content [%] |
| 1 | 5,60 | 1324,2 | 100,00 | 1 | 5,60 | 3424,7 | 100,00 |

Compound **4**

Analytical HPLC was carried out on an Agilent 1200 instrument equipped with a UV-DAD detector. The chromatographic separation was achieved on hexaphenyl coated column, 150mm x 4.6 mm 5 µm (Gemini C6-Phenyl, Phenomenex) using linear gradient at a flow rate of 1.0 mL/min in different phases configurations.

Phases configuration 1

Gemini C6-Phenyl, Phenomenex

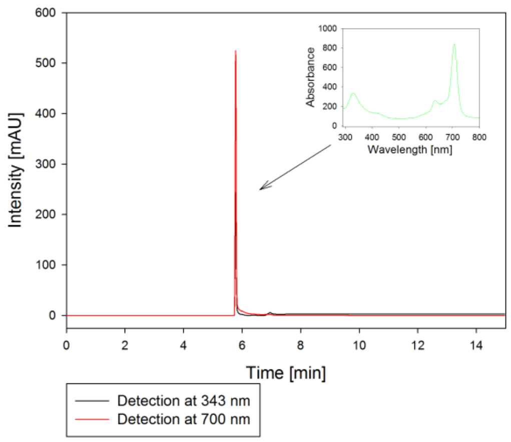


Mobile phase

| Time | $CH_2Cl_2$ | MeOH |
|---|---|---|
| 0 | 100 | 0 |
| 3 | 100 | 0 |
| 4 | 0 | 100 |
| 15 | 0 | 100 |

| Detection at λ = 343 nm | | | | Detection at λ = 700 nm | | | |
|---|---|---|---|---|---|---|---|
| Signal | Retention time [min] | Area | Content [%] | Signal | Retention time [min] | Area | Content [%] |
| 1 | 5,78 | 693,4 | 100,00 | 1 | 5,78 | 1562,6 | 100,00 |

Phases configuration 2

Gemini C6-Phenyl, Phenomenex

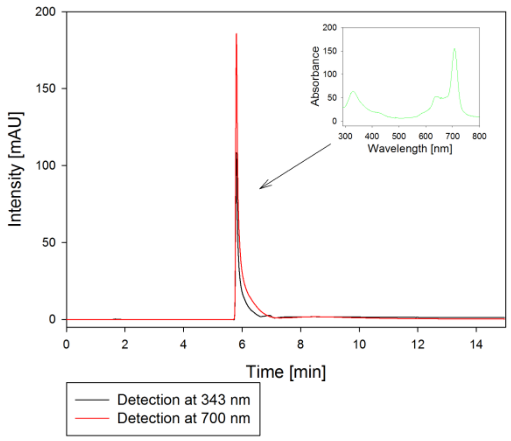


Mobile phase

| Time | $CH_2Cl_2$ | isopropanol |
|---|---|---|
| 0 | 100 | 0 |
| 3 | 100 | 0 |
| 4 | 0 | 100 |
| 15 | 0 | 100 |

| Detection at λ = 343 nm | | | | Detection at λ = 700 nm | | | |
|---|---|---|---|---|---|---|---|
| Signal | Retention time [min] | Area | Content [%] | Signal | Retention time [min] | Area | Content [%] |
| 1 | 5,80 | 945,9 | 100,00 | 1 | 5,80 | 1749,1 | 100,00 |

Phases configuration 3

Gemini C6-Phenyl, Phenomenex

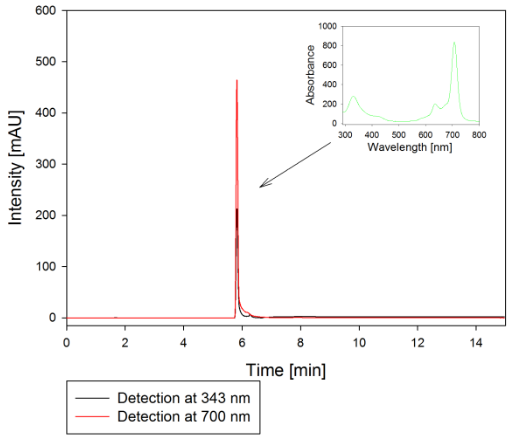

Mobile phase

| Time | $CH_2Cl_2$ | isopropanol | MeOH |
|---|---|---|---|
| 0 | 100 | 0 | 0 |
| 3 | 100 | 0 | 0 |
| 4 | 0 | 50 | 50 |
| 15 | 0 | 50 | 50 |

Detection at λ = 343 nm

| Signal | Retention time [min] | Area | Content [%] |
|---|---|---|---|
| 1 | 5,82 | 1047,8 | 94,98 |
| 2 | 6,27 | 55,4 | 5,02 |

Detection at λ = 700 nm

| Signal | Retention time [min] | Area | Content [%] |
|---|---|---|---|
| 1 | 5,82 | 2338,7 | 100,00 |

Compound **5**

Analytical HPLC was carried out on an Agilent 1200 instrument equipped with a UV-DAD detector. The chromatographic separation was achieved on hexaphenyl coated column, 150mm x 4.6 mm 5 µm (Gemini C6-Phenyl, Phenomenex) using linear gradient conditions at a flow rate of 1.0 mL/min in different configurations.

Phases configuration 1

Gemini C6-Phenyl, Phenomenex

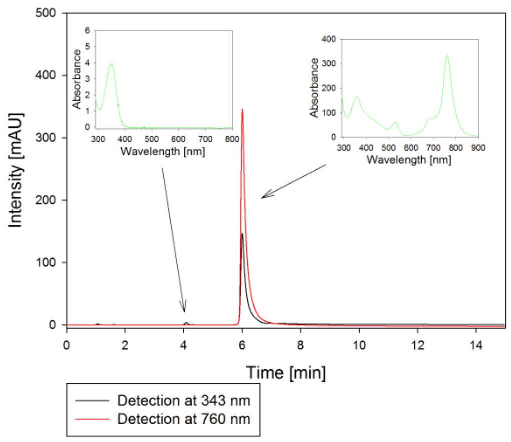


Mobile phase

| Time | MeOH | $H_2O$ |
|---|---|---|
| 0 | 50 | 50 |
| 3 | 50 | 50 |
| 4 | 100 | 0 |
| 15 | 100 | 0 |

| Detection at λ = 343 nm | | | | Detection at λ = 760 nm | | | |
|---|---|---|---|---|---|---|---|
| Signal | Retention time [min] | Area | Content [%] | Signal | Retention time [min] | Area | Content [%] |
| 1 | 1,06 | 9,0 | 0,49 | 1 | 6,01 | 4471,2 | 100,00 |
| 2 | 4,09 | 29,0 | 1,57 | | | | |
| 3 | 6,00 | 1812,8 | 97,94 | | | | |

Phases configuration 2

Gemini C6-Phenyl, Phenomenex

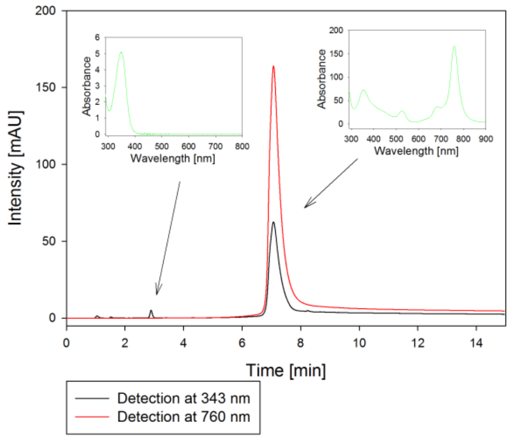


Mobile phase

| Time | MeOH | $H_2O$ |
|---|---|---|
| 0 | 60 | 40 |
| 4 | 60 | 40 |
| 5 | 100 | 0 |
| 15 | 100 | 0 |

Detection at λ = 343 nm

| Signal | Retention time [min] | Area | Content [%] |
|---|---|---|---|
| 1 | 1,05 | 9,6 | 0,62 |
| 2 | 2,89 | 28,1 | 1,83 |
| 3 | 7,07 | 1500,4 | 97,55 |

Detection at λ = 760 nm

| Signal | Retention time [min] | Area | Content [%] |
|---|---|---|---|
| 1 | 7,07 | 4178,5 | 100,00 |

Phases configuration 3

Gemini C6-Phenyl, Phenomenex

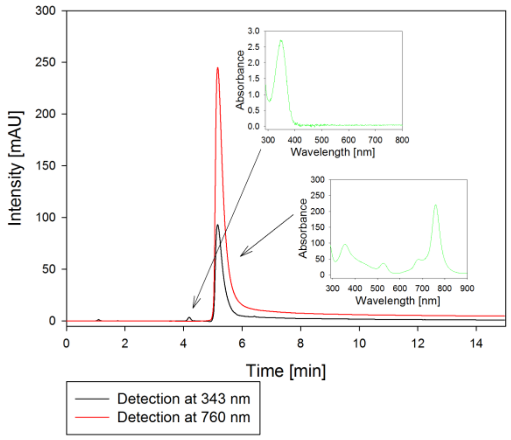


Mobile phase

| Time | MeOH | $H_2O$ |
|---|---|---|
| 0 | 50 | 50 |
| 2 | 50 | 50 |
| 3 | 99 | 1 |
| 15 | 99 | 1 |

Detection at λ = 343 nm

| Signal | Retention time [min] | Area | Content [%] |
|---|---|---|---|
| 1 | 1,10 | 8,2 | 0,45 |
| 2 | 4,19 | 28,0 | 1,55 |
| 3 | 5,16 | 1769,8 | 98,00 |

Detection at λ = 760 nm

| Signal | Retention time [min] | Area | Content [%] |
|---|---|---|---|
| 1 | 5,17 | 5038,9 | 100,00 |

Compound **6**

Phases configuration 1

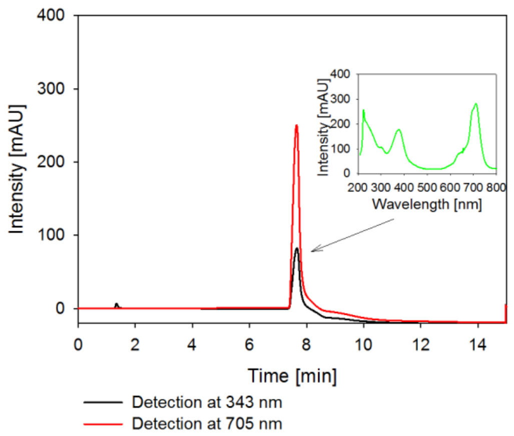


Mobile phase

| Time | MeOH | $CH_2Cl_2$ |
|---|---|---|
| 0 | 95 | 5 |
| 5 | 95 | 5 |
| 6 | 5 | 95 |
| 15 | 5 | 95 |

| | |
|---|---|
| Flow | 1.0 ml/min |
| Temperature | 25°C |
| Column | Agilent. Eclipse XDB-C18 150 mm · 4.6 mm. 5 µm |

Detection at λ=343 nm

| Signal | Retention time [min] | Area | Content [%] |
|---|---|---|---|
| 1 | 1.34 | 40.8 | 2.36 |
| 2 | 7.66 | 1684.6 | 97.64 |

Detection at λ=705 nm

| Signal | Retention time [min] | Area | Content [%] |
|---|---|---|---|
| 1 | 7.65 | 4377.1 | 100 |

Phases configuration 2

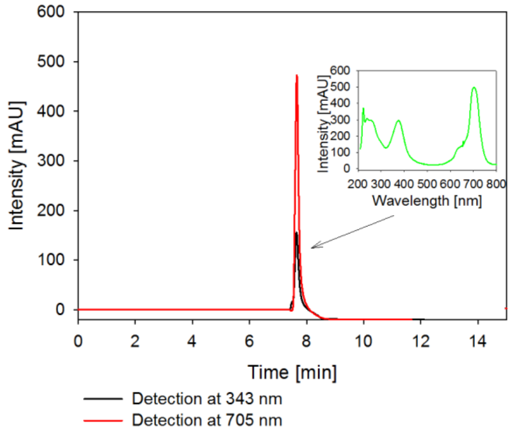


Mobile phase

| Time | MeOH | $CH_2Cl_2$ | 2-propanol |
|---|---|---|---|
| 0 | 5 | 90 | 5 |
| 5 | 5 | 90 | 5 |
| 6 | 45 | 10 | 45 |

| | |
|---|---|
| Flow | 1.0 ml/min |
| Temperature | 25°C |
| Column | Agilent. Eclipse XDB-C18 150 mm · 4.6 mm. 5 µm |

| | | | |
|---|---|---|---|
| 15 | 45 | 10 | 45 |

Detection at λ=343 nm

| Signal | Retention time [min] | Area | Content [%] |
|---|---|---|---|
| 1 | 7.65 | 2002.8 | 100 |

Detection at λ=705 nm

| Signal | Retention time [min] | Area | Content [%] |
|---|---|---|---|
| 1 | 7.65 | 4377.1 | 100 |

# Phases configuration 3

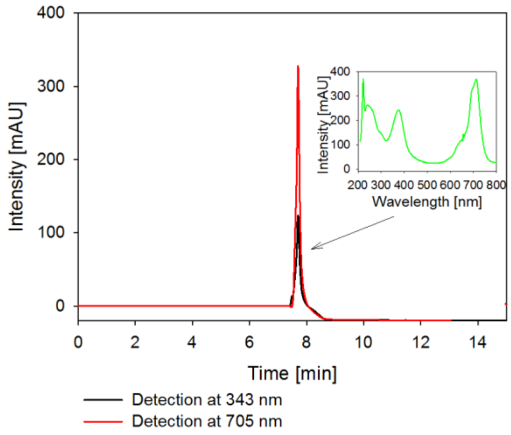


Mobile phase

| Time | MeOH | $CH_2Cl_2$ | ACN |
|---|---|---|---|
| 0 | 98 | 1 | 1 |
| 5 | 98 | 1 | 1 |
| 6 | 94 | 1 | 5 |
| 15 | 94 | 1 | 5 |

| | |
|---|---|
| Flow | 1.0 ml/min |
| Temperature | 25°C |
| Column | Agilent. Eclipse XDB-C18<br>150 mm · 4.6 mm. 5 µm |

Detection at λ=343 nm

| Signal | Retention time [min] | Area | Content [%] |
|---|---|---|---|
| 1 | 7.7 | 1600.8 | 100 |

Detection at λ=705 nm

| Signal | Retention time [min] | Area | Content [%] |
|---|---|---|---|
| 1 | 7.7 | 3229 | 100 |

# Compound **7**

## Phases configuration 1

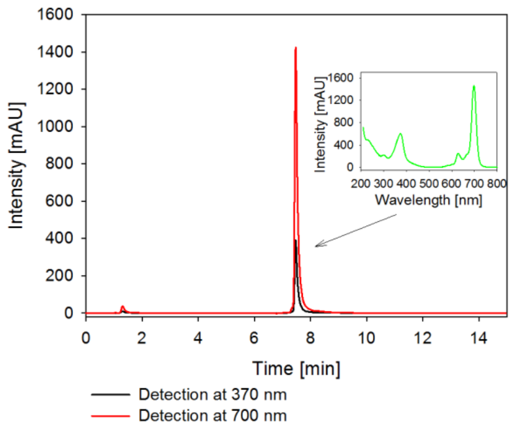


Mobile phase

| Time | MeOH | $H_2O$ |
|---|---|---|
| 0 | 70 | 30 |
| 4 | 70 | 30 |
| 5 | 100 | 0 |
| 15 | 100 | 0 |

| | |
|---|---|
| Flow | 1.0 ml/min |
| Temperature | 25°C |
| Column | Agilent, Eclipse XDB-C18 150 mm · 4.6 mm, 5 µm |

Detection at λ=370 nm

| Signal | Retention time [min] | Area | Content [%] |
|---|---|---|---|
| 1 | 1.3 | 83.09 | 2.71 |
| 2 | 7.47 | 2984.9 | 97.29 |

Detection at λ=700 nm

| Signal | Retention time [min] | Area | Content [%] |
|---|---|---|---|
| 1 | 1.3 | 363.98 | 3.01 |
| 2 | 7.47 | 11720.23 | 96.99 |

## Phases configuration 2

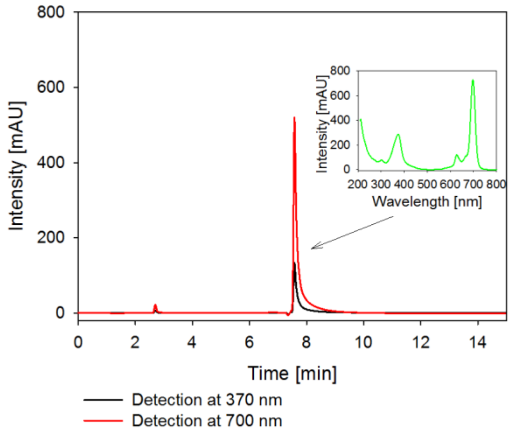


Mobile phase

| Time | MeOH | $H_2O$ |
|---|---|---|
| 0 | 60 | 40 |
| 4 | 60 | 40 |
| 5 | 100 | 0 |
| 15 | 100 | 0 |

| | |
|---|---|
| Flow | 1.0 ml/min |
| Temperature | 25°C |
| Column | Agilent. Eclipse XDB-C18 150 mm · 4.6 mm. 5 µm |

Detection at λ=370 nm

| Signal | Retention time [min] | Area | Content [%] |
|---|---|---|---|
| 1 | 2.7 | 31.19 | 2.11 |
| 2 | 7.58 | 1446.92 | 97.89 |

Detection at λ=700 nm

| Signal | Retention time [min] | Area | Content [%] |
|---|---|---|---|
| 1 | 2.7 | 123.89 | 2.05 |
| 2 | 6.82 | 150.92 | 2.5 |
| 3 | 7.58 | 5765.54 | 95.45 |

# Phases configuration 3

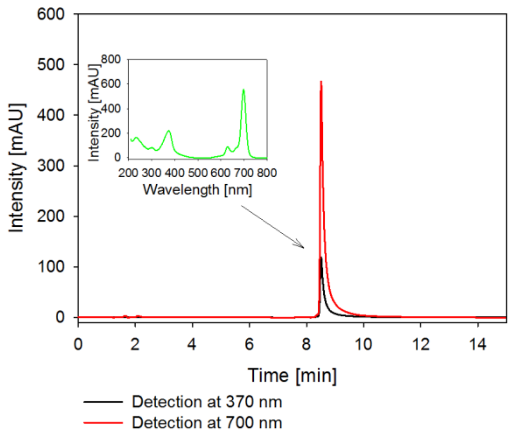


Mobile phase

| Time | MeOH | $H_2O$ | ACN |
|---|---|---|---|
| 0 | 60 | 30 | 10 |
| 5 | 60 | 30 | 10 |
| 6 | 0 | 0 | 100 |
| 15 | 0 | 0 | 100 |

| | |
|---|---|
| Flow | 1.0 ml/min |
| Temperature | 25°C |
| Column | Agilent. Eclipse XDB-C18<br>150 mm · 4.6 mm. 5 µm |

Detection at λ=370 nm

| Signal | Retention time [min] | Area | Content [%] |
|---|---|---|---|
| 1 | 8.51 | 1279.44 | 100 |

Detection at λ=700 nm

| Signal | Retention time [min] | Area | Content [%] |
|---|---|---|---|
| 1 | 1.65 | 29.89 | 0.56 |
| 2 | 2.08 | 49.25 | 0.92 |
| 3 | 8.51 | 5254.71 | 98.52 |

## 5. Computational data

Table S1. Atom coordination of compound **7**.

| number | type | coordinate | | | number | type | coordinate | | |
|---|---|---|---|---|---|---|---|---|---|
| | | x | y | z | | | x | y | z |
| 1 | N | -0.15832000 | -2.0020490 | 0.16467900 | 74 | S | -8.20135300 | -1.0696310 | 0.12129600 |
| 2 | N | 0.15832000 | 2.0020490 | 0.16467900 | 75 | C | -8.89059900 | -0.6115940 | 1.69073500 |
| 3 | N | 2.00204900 | -0.1583200 | 0.16467900 | 76 | N | -8.26154000 | -0.6679610 | 2.85045300 |
| 4 | N | -2.00204900 | 0.1583200 | 0.16467900 | 77 | C | -9.19228100 | -0.2730800 | 3.77777400 |
| 5 | C | -1.34170600 | -2.6975840 | 0.15817900 | 78 | H | -8.94827000 | -0.2125470 | 4.82932900 |
| 6 | C | -0.89999000 | 2.8757930 | 0.15391400 | 79 | C | -10.39394400 | 0.0000000 | 3.17049300 |
| 7 | C | 2.69758400 | -1.3417060 | 0.15817900 | 80 | H | -11.35210200 | 0.3123110 | 3.55719500 |
| 8 | C | 2.87579300 | 0.8999900 | 0.15391400 | 81 | N | -10.19582600 | -0.2073860 | 1.82244400 |
| 9 | C | 1.34170600 | 2.6975840 | 0.15817900 | 82 | C | -11.19264100 | -0.0387350 | 0.77071900 |
| 10 | C | 0.89999000 | -2.8757930 | 0.15391400 | 83 | H | -11.64427400 | -0.9996940 | 0.50237700 |
| 11 | C | -2.69758400 | 1.3417060 | 0.15817900 | 84 | H | -10.73513500 | 0.4152190 | -0.11243600 |
| 12 | C | -2.87579300 | -0.8999900 | 0.15391400 | 85 | H | -11.97590100 | 0.6242550 | 1.14286400 |
| 13 | C | 0.37000400 | -4.2384620 | 0.14073900 | 86 | S | -2.33733400 | -7.9558660 | 0.14452600 |
| 14 | C | 1.03369200 | 4.1253070 | 0.14876600 | 87 | C | -2.23976600 | -8.5787830 | -1.51092400 |
| 15 | C | 4.23846200 | 0.3700040 | 0.14073900 | 88 | N | -1.68262300 | -9.7316400 | -1.84576900 |
| 16 | C | 4.12530700 | -1.0336920 | 0.14876600 | 89 | C | -1.91334600 | -9.8793940 | -3.18764600 |
| 17 | C | -0.37000400 | 4.2384620 | 0.14073900 | 90 | H | -1.56281300 | -10.7462000 | -3.72991100 |
| 18 | C | -1.03369200 | -4.1253070 | 0.14876600 | 91 | C | -2.61935200 | -8.8054850 | -3.67295300 |
| 19 | C | -4.23846200 | -0.3700040 | 0.14073900 | 92 | H | -2.97682400 | -8.5573190 | -4.66099300 |
| 20 | C | -4.12530700 | 1.0336920 | 0.14876600 | 93 | N | -2.84094200 | -7.9780050 | -2.59641700 |
| 21 | N | -2.57818100 | -2.1991880 | 0.14972500 | 94 | C | -3.56441600 | -6.7158230 | -2.62762700 |
| 22 | N | -2.19918800 | 2.5781810 | 0.14972500 | 95 | H | -2.87960600 | -5.8661130 | -2.70185600 |
| 23 | N | 2.19918800 | -2.5781810 | 0.14972500 | 96 | H | -4.15535700 | -6.6136280 | -1.71617200 |
| 24 | N | 2.57818100 | 2.1991880 | 0.14972500 | 97 | H | -4.23449000 | -6.7146570 | -3.48878700 |
| 25 | C | 5.48792900 | 0.9851450 | 0.14979200 | 98 | S | 1.06963100 | -8.2013530 | 0.12129600 |
| 26 | C | 5.26302600 | -1.8387260 | 0.14146500 | 99 | C | 0.61159400 | -8.8905990 | 1.69073500 |
| 27 | C | -0.98514500 | 5.4879290 | 0.14979200 | 100 | N | 0.66796100 | -8.2615400 | 2.85045300 |
| 28 | C | -1.83872600 | -5.2630260 | 0.14146500 | 101 | C | 0.27308000 | -9.1922810 | 3.77777400 |
| 29 | C | -5.48792900 | -0.9851450 | 0.14979200 | 102 | H | 0.21254700 | -8.9482700 | 4.82932900 |
| 30 | C | -5.26302600 | 1.8387260 | 0.14146500 | 103 | C | 0.00000000 | -10.3939440 | 3.17049300 |
| 31 | C | 0.98514500 | -5.4879290 | 0.14979200 | 104 | H | -0.31231100 | -11.3521020 | 3.55719500 |
| 32 | C | 1.83872600 | 5.2630260 | 0.14146500 | 105 | N | 0.20738600 | -10.1958260 | 1.82244400 |
| 33 | C | 6.63705200 | 0.1878850 | 0.14599700 | 106 | C | 0.03873500 | -11.1926410 | 0.77071900 |
| 34 | C | 6.52249000 | -1.2330800 | 0.11284200 | 107 | H | 0.99969400 | -11.6442740 | 0.50237700 |
| 35 | C | -0.18788500 | 6.6370520 | 0.14599700 | 108 | H | -0.41521900 | -10.7351350 | -0.11243600 |
| 36 | C | -1.23308000 | -6.5224900 | 0.11284200 | 109 | H | -0.62425500 | -11.9759010 | 1.14286400 |
| 37 | C | -6.63705200 | -0.1878850 | 0.14599700 | 110 | S | -7.95586600 | 2.3373340 | 0.14452600 |
| 38 | C | -6.52249000 | 1.2330800 | 0.11284200 | 111 | C | -8.57878300 | 2.2397660 | -1.51092400 |
| 39 | C | 0.18788500 | -6.6370520 | 0.14599700 | 112 | N | -9.73164000 | 1.6826230 | -1.84576900 |
| 40 | C | 1.23308000 | 6.5224900 | 0.11284200 | 113 | C | -9.87939400 | 1.9133460 | -3.18764600 |
| 41 | Mg | 0.00000000 | 0.0000000 | 0.19856800 | 114 | H | -10.74620000 | 1.5628130 | -3.72991100 |
| 42 | H | -2.06466300 | 5.5754910 | 0.18870800 | 115 | C | -8.80548500 | 2.6193520 | -3.67295300 |
| 43 | H | 2.91903600 | 5.1730720 | 0.15143700 | 116 | H | -8.55731900 | 2.9768240 | -4.66099300 |
| 44 | H | 5.57549100 | 2.0646630 | 0.18870800 | 117 | N | -7.97800500 | 2.8409420 | -2.59641700 |
| 45 | H | 5.17307200 | -2.9190360 | 0.15143700 | 118 | C | -6.71582300 | 3.5644160 | -2.62762700 |
| 46 | H | 2.06466300 | -5.5754910 | 0.18870800 | 119 | H | -5.86611300 | 2.8796060 | -2.70185600 |
| 47 | H | -2.91903600 | -5.1730720 | 0.15143700 | 120 | H | -6.61362800 | 4.1553570 | -1.71617200 |
| 48 | H | -5.57549100 | -2.0646630 | 0.18870800 | 121 | H | -6.71465700 | 4.2344900 | -3.48878700 |
| 49 | H | -5.17307200 | 2.9190360 | 0.15143700 | 122 | S | 7.95586600 | -2.3373340 | 0.14452600 |
| 50 | S | -1.06963100 | 8.2013530 | 0.12129600 | 123 | C | 8.57878300 | -2.2397660 | -1.51092400 |
| 51 | C | -0.61159400 | 8.8905990 | 1.69073500 | 124 | N | 9.73164000 | -1.6826230 | -1.84576900 |
| 52 | N | -0.66796100 | 8.2615400 | 2.85045300 | 125 | C | 9.87939400 | -1.9133460 | -3.18764600 |
| 53 | C | -0.27308000 | 9.1922810 | 3.77777400 | 126 | H | 10.74620000 | -1.5628130 | -3.72991100 |
| 54 | H | -0.21254700 | 8.9482700 | 4.82932900 | 127 | C | 8.80548500 | -2.6193520 | -3.67295300 |
| 55 | C | 0.00000000 | 10.3939440 | 3.17049300 | 128 | H | 8.55731900 | -2.9768240 | -4.66099300 |
| 56 | H | 0.31231100 | 11.3521020 | 3.55719500 | 129 | N | 7.97800500 | -2.8409420 | -2.59641700 |
| 57 | N | -0.20738600 | 10.1958260 | 1.82244400 | 130 | C | 6.71582300 | -3.5644160 | -2.62762700 |
| 58 | C | -0.03873500 | 11.1926410 | 0.77071900 | 131 | H | 5.86611300 | -2.8796060 | -2.70185600 |
| 59 | H | -0.99969400 | 11.6442740 | 0.50237700 | 132 | H | 6.61362800 | -4.1553570 | -1.71617200 |
| 60 | H | 0.41521900 | 10.7351350 | -0.11243600 | 133 | H | 6.71465700 | -4.2344900 | -3.48878700 |
| 61 | H | 0.62425500 | 11.9759010 | 1.14286400 | 134 | S | 8.20135300 | 1.0696310 | 0.12129600 |
| 62 | S | 2.33733400 | 7.9558660 | 0.14452600 | 135 | C | 8.89059900 | 0.6115940 | 1.69073500 |
| 63 | C | 2.23976600 | 8.5787830 | -1.51092400 | 136 | N | 8.26154000 | 0.6679610 | 2.85045300 |
| 64 | N | 1.68262300 | 9.7316400 | -1.84576900 | 137 | C | 9.19228100 | 0.2730800 | 3.77777400 |
| 65 | C | 1.91334600 | 9.8793940 | -3.18764600 | 138 | H | 8.94827000 | 0.2125470 | 4.82932900 |
| 66 | H | 1.56281300 | 10.7462000 | -3.72991100 | 139 | C | 10.39394400 | 0.0000000 | 3.17049300 |

| | | | | | | | | | |
|---|---|---|---|---|---|---|---|---|---|
| 67 | C | 2.61935200 | 8.8054850 | -3.67295300 | 140 | H | 11.35210200 | -0.3123110 | 3.55719500 |
| 68 | H | 2.97682400 | 8.5573190 | -4.66099300 | 141 | N | 10.19582600 | 0.2073860 | 1.82244400 |
| 69 | N | 2.84094200 | 7.9780050 | -2.59641700 | 142 | C | 11.19264100 | 0.0387350 | 0.77071900 |
| 70 | C | 3.56441600 | 6.7158230 | -2.62762700 | 143 | H | 11.64427400 | 0.9996940 | 0.50237700 |
| 71 | H | 2.87960600 | 5.8661130 | -2.70185600 | 144 | H | 10.73513500 | -0.4152190 | -0.11243600 |
| 72 | H | 4.15535700 | 6.6136280 | -1.71617200 | 145 | H | 11.97590100 | -0.6242550 | 1.14286400 |
| 73 | H | 4.23449000 | 6.7146570 | -3.48878700 | 74 | S | -8.20135300 | -1.0696310 | 0.12129600 |

Summary of NMR spectra ( SCF GIAO Magnetic shielding), Values of **7** for element C only
Degenerate peaks are condensed together (Degeneracy Tolerance 0.05)

| # Shielding (ppm) | Degeneracy | Atoms |
|---|---|---|
| 22.4243000000 | 4.0000 | 5,7,9,11 |
| 22.7236000000 | 4.0000 | 6,8,10,12 |
| 29.7097000000 | 4.0000 | 33,35,37,39 |
| 30.3117000000 | 4.0000 | 51,75,99,135 |
| 34.3942000000 | 4.0000 | 63,87,111,123 |
| 34.8692000000 | 4.0000 | 34,36,38,40 |
| 36.1343000000 | 4.0000 | 13,15,17,19 |
| 36.6794000000 | 4.0000 | 14,16,18,20 |
| 43.2229000000 | 4.0000 | 26,28,30,32 |
| 44.5690000000 | 4.0000 | 25,27,29,31 |
| 48.6029000000 | 4.0000 | 53,77,101,137 |
| 49.4615000000 | 4.0000 | 65,89,113,125 |
| 51.5323000000 | 4.0000 | 67,91,115,127 |
| 53.8700000000 | 4.0000 | 55,79,103,139 |
| 147.1335500000 | 8.0000 | 58,82,106,142,70,94,118,130 |

# 6. Crystallographic data

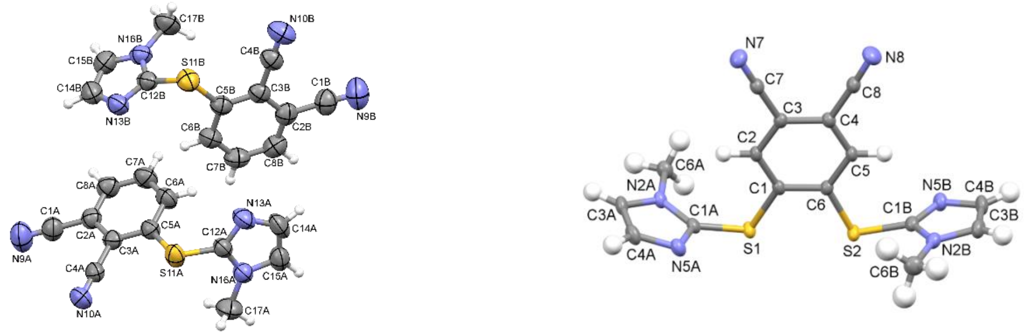


Figure S1. Anisotropic ellipsoid representation of **1** (left) and **2** (right), showing the atom-labeling scheme**.** Displacement ellipsoids are drawn at the 50% probability level and H atoms are shown as small spheres of arbitrary radii.

7. Liposomes

Table S2. Size of liposomal formulation of studied phthalocyanines

| Compound | Mean diameter [nm] | Dv10 | Dv50 | Dv90 |
|---|---|---|---|---|
| **3** | 152 | 94 | 131 | 248 |
| **4** | 155 | 100 | 148 | 199 |
| **5** | 265 | 133 | 240 | 422 |
| **6** | 141 | 85 | 139 | 191 |
| **7** | 160 | 110 | 155 | 200 |

8. Dark activity of PSs against *Staphylococcus aureus*

Table S3. Dark activity of PSs against *Staphylococcus aureus*

| Compound | **3** | **4** | **5** | **6** | **7** |
|---|---|---|---|---|---|
| Concentration [M] | log reduction in bacterial growth | | | | |
| $10^{-4}$ | 0.08 | 0.00 | 0.06 | 0.20 | 0.08 |
| $10^{-5}$ | 0.02 | 0.07 | 0.03 | 0.11 | 0.11 |
